# Phosphorylations of the Abutilon Mosaic Virus Movement Protein Affect Its Self-Interaction, Symptom Development, Viral DNA Accumulation, and Host Range

**DOI:** 10.3389/fpls.2020.01155

**Published:** 2020-07-31

**Authors:** Tatjana Kleinow, Andrea Happle, Sigrid Kober, Luise Linzmeier, Tina M. Rehm, Jacques Fritze, Patrick C. F. Buchholz, Gabi Kepp, Holger Jeske, Christina Wege

**Affiliations:** Institute of Biomaterials and Biomolecular Systems, University of Stuttgart, Stuttgart, Germany

**Keywords:** geminivirus transport, host plant-virus interaction, plant-derived posttranslational modification, mutagenesis, Förster resonance energy transfer (FRET)

## Abstract

The genome of bipartite geminiviruses in the genus *Begomovirus* comprises two circular DNAs: DNA-A and DNA-B. The DNA-B component encodes a nuclear shuttle protein (NSP) and a movement protein (MP), which cooperate for systemic spread of infectious nucleic acids within host plants and affect pathogenicity. MP mediates multiple functions during intra- and intercellular trafficking, such as binding of viral nucleoprotein complexes, targeting to and modification of plasmodesmata, and release of the cargo after cell-to-cell transfer. For Abutilon mosaic virus (AbMV), phosphorylation of MP expressed in bacteria, yeast, and *Nicotiana benthamiana* plants, respectively, has been demonstrated in previous studies. Three phosphorylation sites (T221, S223, and S250) were identified in its C-terminal oligomerization domain by mass spectrometry, suggesting a regulation of MP by posttranslational modification. To examine the influence of the three sites on the self-interaction in more detail, MP mutants were tested for their interaction in yeast by two-hybrid assays, or by Förster resonance energy transfer (FRET) techniques *in planta*. Expression constructs with point mutations leading to simultaneous (triple) exchange of T221, S223, and S250 to either uncharged alanine (MP^AAA^), or phosphorylation charge-mimicking aspartate residues (MP^DDD^) were compared. MP^DDD^ interfered with MP-MP binding in contrast to MP^AAA^. The roles of the phosphorylation sites for the viral life cycle were studied further, using plant-infectious AbMV DNA-B variants with the same triple mutants each. When co-inoculated with wild-type DNA-A, both mutants infected *N. benthamiana* plants systemically, but were unable to do so for some other plant species of the families Solanaceae or Malvaceae. Systemically infected plants developed symptoms and viral DNA levels different from those of wild-type AbMV for most virus-plant combinations. The results indicate a regulation of diverse MP functions by posttranslational modifications and underscore their biological relevance for a complex host plant-geminivirus interaction.

## Introduction

Geminiviruses constitute an economically important group of plant-infecting viruses (Rojas et al., 2018; Garcia-Arenal and Zerbini, 2019). Their genomes consist of either one or two (DNA-A & DNA-B) circular single-stranded (ss) DNA molecules, which are packaged into uniquely shaped “geminate”, i.e., double-icosahedral particles (Jeske, 2009; Hanley-Bowdoin et al., 2013; Hipp et al., 2017; Hesketh et al., 2018; Xu et al., 2019). Within nuclei of plant host cells, viral DNAs (vDNAs) replicate *via* double-stranded (ds) intermediates that adopt minichromosome organization (Pilartz and Jeske, 1992; Pilartz and Jeske, 2003; Jeske, 2009; Hanley-Bowdoin et al., 2013).

Based on the currently available data, it can be assumed that geminiviruses have developed manifold and redundant strategies for transferring newly replicated vDNA from the nucleus towards the cell periphery and across plasmodesmata into adjacent cells, as well as for vDNA long-distance spread *via* phloem tissues (Rojas et al., 2005; Wege, 2007; Jeske, 2009; Krenz et al., 2012; Hanley-Bowdoin et al., 2013). Depending on the gene composition within mono- or bipartite genomes of Old and New World geminiviruses, the portfolio of viral proteins mediating intra- and intercellular transport may vary (Jeske, 2009; Hanley-Bowdoin et al., 2013). It is generally accepted that monopartite geminiviruses utilize their coat protein (CP; V1) to shuttle vDNA between nuclei and cytoplasm, whereas bipartite members (in the genus *Begomovirus*) mainly employ the DNA-B-encoded nuclear shuttle protein NSP (NSP; BV1) for this task. Subsequent cytoplasmic trafficking of the nucleoprotein complexes towards and through plasmodesmata (PD) might be mediated primarily either by the pre-coat protein (PCP; [A]V2) in especially in monopartite virus species, or by the DNA-B encoded movement protein (MP; BC1) in bipartite ones. Among those, only Old World members express a PCP that could take over certain functions in cell-to-cell spread (Jeske, 2009). The PCP may be assisted in its action by the (A)C4 protein (Jupin et al., 1994; Rojas et al., 2001; Teng et al., 2010). Due to the lack of an *AV2* gene, begomoviruses of the New World, such as the Abutilon mosaic virus (AbMV), rely on their MP for intra- and intercellular trafficking. The nuclear shuttle function can be mediated by NSP or redundantly by CP in case of a defective NSP (Qin et al., 1998). Besides their coordinated action during viral spread, NSP and MP determine viral pathogenicity collaboratively (Rojas et al., 2005; Zhou et al., 2007; Jeske, 2009; Hanley-Bowdoin et al., 2013).

Geminivirus genomes have a limited coding capacity (five to eight genes) causing a strong dependency on host cellular processes and factors for propagation and systemic invasion of host tissues. The viral proteins fulfill multiple functions and interact in a highly coordinated manner with a variety of plant proteins. The membrane-associated MPs of bipartite begomoviruses are prime examples in this regard. They enable for instance the targeting of viral transport complexes to PD, modify PDs in order to allow passage, and release the infectious cargo after successful cell-to-cell transfer (Jeske, 2009; Krenz et al., 2012; Hanley-Bowdoin et al., 2013). Some MP interacting host proteins have been discovered: histone H3 (Zhou et al., 2011), a synaptotagmin (Lewis and Lazarowitz, 2010), and a chaperone, i.e., the heat shock cognate 70kDa protein cpHSC70-1 (Krenz et al., 2010). Such multifaceted properties do not only apply to geminiviral proteins, but are characteristic of many membrane-targeted proteins of various virus species that can frequently self-interact, and bind other viral proteins and host factors (Laliberte and Sanfacon, 2010; Laliberte and Zheng, 2014).

**Figure 1:**
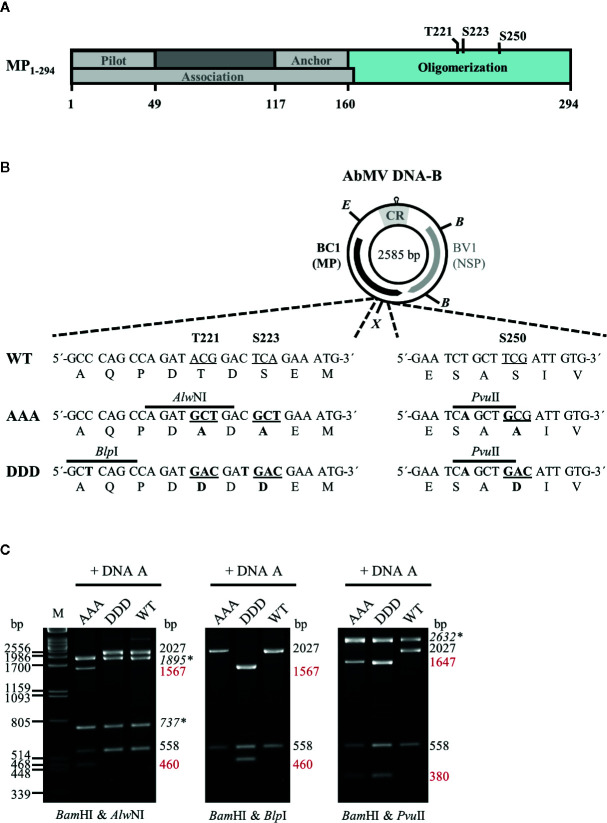
**(A)** Schematic overview of previously described functional regions and phosphorylation sites identified within full-length AbMV MP (Zhang et al., 2001; Frischmuth et al., 2004; Kleinow et al., 2009b; Krenz et al., 2010; Krapp et al., 2017). **(B)** Schematic representation of the organization of DNA-B encoding the movement protein (MP; BC1) and the nuclear shuttle protein (NSP; BV1). *Eco *RV (E) and *Xba *I (X) sites used for cloning of DNA-B versions with triple phosphorylation site mutations [T221, S223, and S250 exchanged against alanine (AAA) or aspartate (DDD)] for MP are indicated. Details of the site-directed mutagenesis (nucleotide and amino acid changes shown in bold) and newly introduced mutagenesis-specific restriction sites are depicted below. CR: common region; B: *Bam *HI **(C)** Representative RCA-RFLP analysis using *Bam *HI combined with either of the mutagenesis-specific restriction enzymes to monitor the presence of DNA-A and DNA-B variants within total nucleic acids from infected plants (Kleinow et al., 2009b). Sizes of expected fragments are indicated. DNA-A-derived fragments are labelled (italics and asterisks), with those specifically originating from mutagenesis within *BC1* displayed in red. RCA-RFLP analysis was carried out for all samples out of the experiments listed in **Tables 1** and **2**.

For AbMV MP, selected as a model in our study, several of these features have been described. MP and NSP cooperate for intra- and intercellular traffic (Zhang et al., 2001). Self-interaction of MP *via* its C-terminal domain was demonstrated in yeast systems (Frischmuth et al., 2004), and for the full-length MP *in planta* by bimolecular fluorescence complementation (BiFC) and Förster resonance energy transfer (FRET) techniques (Happle et al., submitted; Krenz et al., 2010). Membrane association is mediated by the central part of the protein (named anchor domain; **Figure 1A**) as uncovered *in planta* by studying the subcellular localization of green fluorescent protein (GFP)-tagged MP deletion mutants (Zhang et al., 2002). Computer-based analysis predicted an amphiphilic helix structure for this domain, suggesting the MP is a monotopic membrane protein with N- and C-termini located at the cytoplasmic membrane face, as shown in fission yeast by freeze-fracture immunolabeling in both the plasma membrane and microsomes (Aberle et al., 2002; Frischmuth et al., 2004). High resolution fluorescence and electron microscopy-based analysis of either fluorescent protein- or epitope-tagged MP confirmed its localization at the plasma membrane and plasmodesmata of plant cells (Happle et al., submitted; Zhang et al., 2001; Kleinow et al., 2009c). Additionally, the MP was found surrounding nuclei, associated with chloroplasts, and clustered in mobile vesicle-like structures, with the latter most likely representing microsomes trafficking *via* the endoplasmic reticulum (ER)/actin network (Happle et al., submitted; Zhang et al., 2001; Krenz et al., 2010).

Upon systemic infection of a permissive host, vDNA passes through various stages of replication, transcription, transport, and encapsidation, which need to be temporally organized. It can be expected that in the course of these processes, the viral MPs need to switch between several functional options in order to regulate their subcellular distribution and control distinct protein-protein interactions. Posttranslational modifications (PTMs) and especially sequence-specific and reversible phosphorylation play important regulatory roles in many processes in plants, e.g. in subcellular localization, stability and interaction of proteins, metabolic regulation, defense signal transduction, RNA and carbon metabolism (Link et al., 2011; Burch-Smith and Zambryski, 2012; Lofke et al., 2013; Nemes et al., 2017; Ott, 2017; Arsova et al., 2018; Perraki et al., 2018; Zhukovsky et al., 2019). Accordingly, numerous examples of specific PTMs were uncovered to regulate plant endogenous macromolecular trafficking and viral spread (Lee and Lucas, 2001; Waigmann et al., 2004; Lee et al., 2005; Taoka et al., 2007; Amari et al., 2010). Phosphorylation is the most widespread one, identified in proteins of distinct plant DNA- and RNA-viruses. Several MPs of the 30K family (Melcher, 2000), to which the geminiviral MPs belong, were shown to appear as phosphoproteins *in planta* (Waigmann et al., 2004; Kleinow et al., 2009b; Nemes et al., 2017; and references therein). Well studied examples in the 30K family are MPs of the tobamoviruses tobacco mosaic virus (TMV) and tomato mosaic virus (ToMV), for which roles of phosphorylation in modulation of RNA affinity, subcellular distribution, membrane association, plasmodesmata targeting, and transport activity have been delimited (Karpova et al., 1999; Kawakami et al., 1999; Karger et al., 2003; Waigmann et al., 2004; Trutnyeva et al., 2005; Fujiki et al., 2006). For bipartite begomoviruses, however, studies on the incidence of PTMs and their putative regulatory impact on MPs are scarce. A phosphorylation of MPs was uncovered by metabolic labelling experiments for squash leaf curl virus in insect cells (Pascal et al., 1994), for AbMV in bacteria and yeast (Wege and Jeske, 1998; Kleinow et al., 2008) and by *in vitro* phosphorylation assays using a plasmodesmal-associated protein kinase for bean dwarf mosaic virus (BDMV) (Lee et al., 2005). In addition, phosphorylation on serine and threonine residues *in planta* could be shown for AbMV MP by Western blot analysis using phospho-amino acid-specific antibodies (Kleinow et al., 2009b). Follow-up work identified three phosphorylation sites, T221, S223, and S250 (**Figure 1A**) within the oligomerization domain of AbMV MP that are each differentially important for symptom development and vDNA accumulation in *Nicotiana benthamiana* Domin (Kleinow et al., 2009b), indicating a functional role of the phosphorylation status at these positions, and a possible effect on intra- and intercellular spread of the virus. The biological relevance of the three sites was examined by introducing point mutations into the MP gene of infectious DNA-B clones, in order to compare the “constant-off of charge” (replacement by uncharged alanine residues) or the “constant-on of charge” status of the negative charges (replacement by aspartate residues) with the regulated wild-type (WT) function for the three MP amino acid positions (Kleinow et al., 2009b).

In the previously described experiments (Kleinow et al., 2009b), single and double mutants were compared. Five out of the six tested combinations of DNA-A with DNA-B variants for T221 and/or S223 enhanced symptoms, and three out of these five elevated the steady-state vDNA content as well. Only the combination of DNA-A & DNA-B S223A did not alter symptoms considerably in comparison to AbMV^WT^, although the vDNA level was reduced. At amino acid position S250, both mutations attenuated symptom development and reduced the vDNA titer. Overall, the results showed that the respective phosphorylation sites may trigger opposite effects, with intensifying (T221 and S223) or weakening (S250) AbMV pathogenicity.

To further examine the complexity of the differential MP phosphorylation, we have generated AbMV DNA-Bs encoding MPs with triple mutations of the phosphorylation sites, and analyzed their effects on systemic virus infections in plants. In order to detect possible host-dependent differences, a number of plant species has been included. The localization of the mutations within the MP oligomerization domain may suggest an impact of the phosphorylation status on self-interaction, which was analyzed using the yeast two-hybrid system and a FRET measurement approach *in planta*.

## Materials and Methods

### Cloning Procedure

AbMV DNA-B variants encoding triple phosphorylation site mutations of MP (Uniprot P21946) [T221A/S223A/S250A (MP^AAA^) or T221D/S223D/S250D (MP^DDD^)] were generated by a Phi29 polymerase-based rolling circle amplification (RCA) strategy and thereby a cell-free cloning approach, which preserves the quasi-species nature of geminiviral DNAs (Jeske et al., 2010; Jeske, 2018). DNA-Bs encoding single or double phosphorylation site MP mutants (T221A/S223A, S250A, T221D/S223D or S250D) (Kleinow et al., 2009b) were amplified by RCA from total nucleic acids isolated *via* a CTAB-based extraction protocol (2.3) from *N. benthamiana* systemically infected with the respective AbMV mutant version (Kleinow et al., 2009b). The RCA products were cut by *Eco *RV and *Xba *I according to supplier´s recommendation (New England Biolabs, Ipswich, MA, USA). The resulting two 1,726 bp-fragments comprising mutated sites T221A/S223A or T221D/S223D, and the two 859 bp-fragments including the position encoding for S250A or S250D (**Figure 1**) were gel-purified. To create MP^AAA^- or MP^DDD^-DNA-B variants, the DNA fragments harboring double mutations at T221 and S223 were ligated with those comprising the appropriate S250 mutant. For purification of the desired DNA-B types, molecules circularized by ligation were RCA-amplified and digested with the mutant-specific restriction enzyme *Pvu *II (**Figure 1**). Linearized DNA-B fragments were gel-purified, again re-ligated to circular DNAs and amplified by RCA. The correctness of the obtained DNA-B variants was confirmed by restriction analysis using *Bam* HI in combination with either of the three mutant-specific restriction enzymes (*Alw *NI, *Blp *I and *Pvu *II; **Figure 1**) and direct sequencing of RCA products (Schubert et al., 2007). DNA-Bs confirmed to encode either MP^AAA^ or MP^DDD^ were used for biolistic plant inoculation (2.2).

Two-hybrid expression constructs were obtained by Gateway cloning (ThermoFisher Scientific/Invitrogen, Carlsbad, CA, USA). To create entry vectors containing partial *MP* genes coding for the oligomerization domain (BC1-CD; **Figure 1A**) with triple exchanges at positions T221, S223, and S250 against either aspartate (DDD) or alanine (AAA) residues, a PCR-based site-directed mutagenesis strategy using back-to-back orientated primers according to Kleinow et al. (2009b) was applied. Briefly, in each of the various primer pairs used for amplification, one primer introduced the intended mutation and an additional unique restriction site for selection (**Figure 1B**) (Kleinow et al., 2009b). The vector pENTR11-BC1-CD^WT^ (**Supplementary Table S1**) was subjected to two rounds of mutagenesis PCR (a first round introducing changes at positions T221 and S223, and a second one for additional replacement at amino acid position S250) to yield triple mutants. All PCR products were gel-purified, their 5´-ends phosphorylated, re-ligated to obtain circular plasmids and transformed into *Escherichia coli* (DH5α; ThermoFisher Scientific/Invitrogen). Plasmid DNAs from the resulting clones were subjected to mutant-specific restriction analysis and sequencing. From correct entry constructs containing *BC1-CD^DDD^* or *BC1-CD^AAA^*, these DNA fragments were recombined by LR reactions into the Gateway-compatible yeast two hybrid vectors pcACT2 (Leu^+^; N-terminal fusion to Gal4 activation domain [GAD]::hemagglutinin epitope (HA) yielding GAD::HA::BC1-CD^DDD/AAA^) and pAS2-1 (Trp^+^; N-terminal fusion to Gal4 DNA binding domain [GBD] yielding GBD::BC1-CD^DDD/AAA^). Other two-hybrid constructs used in this study were described previously (Kleinow, 2000; Frischmuth et al., 2004).

*In planta* expression constructs of MP variants for analyzing subcellular co-localization and protein interactions by FRET were prepared *via* a Gateway recombination-based two-in-one-vector system (Hecker et al., 2015). Genes encoding full-length MP^WT^, MP^AAA^ or MP^DDD^ from respective pDONR207-BC1 templates were PCR-amplified (1 min at 98°C, 30 cycles of 10 s at 98°C, 30 s at 54°C, and 1 min at 72°C followed by 5 min at 72°C) using Q5 polymerase (New England Biolabs) with primer pairs adding either attB1 & attB4, or attB3 & attB2 sites, respectively, to produce ends applicable in the Gateway recombination system (ThermoFisher Scientific/Invitrogen) (for templates and primers see **Supplementary Table S1**). All ORFs were amplified in two versions, i.e., with and without stop codon to allow later expression in N- as well as C-terminal fusion to a fluorescent reporter protein. The four different PCR products per MP type were purified (PCR purification kit, Qiagen, Hilden, Germany) and introduced *via* a BP reaction into the compatible pDONR221 vector (pDONR221-P1P4 for attB1 and attB4-containing fragments, or pDONR221-P3P2 for attB3- and attB2-harboring fragments) (Hecker et al., 2015) according to manufacturer´s recommendations (ThermoFisher Scientific/Invitrogen). The various entry constructs were confirmed by restriction analysis and sequencing. Verified *MP* genes were transferred by LR reactions into the compatible binary FRET expression vectors (molar ratio of vector to entry clones of 1:3:3; Grefen and Blatt, 2012; Hecker et al., 2015). The vectors allow from one T-DNA dual expression of the test proteins in four different combinations of either N- or C-terminal fusions to the FRET fluorophore pair monomeric Venus (mVenus; acceptor) and monomeric Turquoise2 (donor) (pFRETtv-2-in-1 -NN: both N-terminal, pFRETtv-2-in-1 -NC: C-terminal mVenus & N-terminal mTurquoise2, pFRETtv-2-in-1 -CN: N-terminal mVenus & C-terminal mTurquoise2 or pFRETtv-2-in-1 -CC: both C-terminal). After confirmation by restriction analysis and sequencing, the resulting FRET expression constructs were introduced into chemically competent *Agrobacterium tumefaciens* GV3101 (pMP90) (Koncz et al., 1994) *via* transformation. Construct integrity in agrobacteria was rechecked by colony PCR.

### Plant Material and Infection

Plants of *N. benthamiana*, *Malva parviflora* L., *Datura stramonium* L., and *Nicandra physaloides* L. were cultivated in an insect-free biosafety S2 containment greenhouse with supplementary lighting (100 kW/h, 16 h photoperiod at 25°C and a night-time reduction to 20°C) at 60% rel. humidity.

Plants were inoculated with RCA products of DNA-A mixed with RCA products of the respective DNA-Bs (2.1) in a four leaf-stage using particle bombardment as described previously (Kleinow et al., 2009b). AbMV DNA-A (X15984) RCA products for inoculation were amplified from *N. benthamiana* plants confirmed to be systemically infected with DNA-A alone following agro-inoculation (Evans and Jeske, 1993; Kleinow et al., 2009b). Plants inoculated with either gold microcarriers devoid of DNA, or with DNA-B^WT^ alone served as mock-treated controls. All RCA inocula applied in this study were generated using the same template and polymerase source, and were prepared in sufficient amounts to conduct at minimum two completely independent infection experiments per batch. Each inoculum batch was verified by RCA-restriction fragment length polymorphism (RCA-RFLP) testing and direct sequencing (2.4). In total, nine independent experiments were carried out for *N. benthamiana*, and for the other plant species at minimum four experiments (**Tables 1** and **2**). For the host *N. benthamiana*, RCA products from plants systemically infected with AbMV variants encoding either MP^AAA^ or MP^DDD^ were passaged by biolistic inoculation to a secondary set of *N. benthamiana* (four plants per selected sample). Three independent viral progenies per AbMV triple mutant type were subjected to these secondary infection experiments. All plants were evaluated for symptom development weekly and tested for infection with the proper AbMV variant as described below (2.3 and 2.4).

**Table 1:** Infectivity and symptom phenotype classification of AbMV DNA-B encoded MP phosphorylation site mutants in *N. benthamiana*.

AbMV DNA-B variant^i^	No. of plants (8 expts.)		Infection rate (%)	Symptom class (no. of plants)^iii^				
	Inoculated	Infected		0	I	II	III	IV
Mock-treated	32	0	0					
WT	41^ii^	41	100	0	0	41	0	0
T221A/S223A^iv^	26	20	77	0	1	4	4	11
S250A^iv^	27	18	67	0	12	4	2	0
T221A/S223A/S250A	54^ii^	52	96	40	12	0	0	0
T221D/S223D^iv^	30	22	73	0	3	2	2	15
S250D^iv^	27	21	78	0	13	3	4	1
T221D/S223D/S250D	54^ii^	51	94	0	2	31	8	10

^i^DNA-B variants were co-inoculated with AbMV DNA-A^WT^.

^ii^Including an additional ninth passage experiment (virus progenies from three independent primary-inoculated plants were used as inoculum for a secondary set of plants [for each inoculum four plants]); per triple mutant variant in total 42 primary (eight experiments [expts.]) and 12 secondary-inoculated plants (one expt.), and for WT in total 29 primary- (eight expts.) and 12 secondary-inoculated plants (one expt.).

^iii^AbMV symptoms (stunting, yellow-green leaf mosaic and leaf deformation) were classified as follows: class 0: all features significantly milder than WT, nearly symptomless; class I: one to two features significantly milder than WT; class II: similar to WT; class III: at least one feature significantly more intense than WT; class IV: two to three features significantly more severe than WT.

^iv^Single and double mutants used as a reference were characterized previously in Kleinow et al. (2009b).

**Table 2:** Infectivity of AbMV DNA-B encoding MP phosphorylation site mutants in various host plants.

Host plant species	No of expts.	AbMV DNA-B variant^i^	No. of plants		Infection rate (%)
			Inoculated	Infected	
*Nicotiana benthamiana*	9	Mock-treated	32	0	0
(Solanaceae)		WT	41	41	100
		AAA	54	52	96
		DDD	54	51	94
*Malva parviflora*	5	Mock-treated	15	0	0
(Malvaceae)		WT	67	37	55
		AAA	188	0^ii^	0^ii^
		DDD	108	24	22
*Datura stramonium*	5	Mock-treated	15	0	0
(Solanaceae)		WT	45	20	44
		AAA	66	27	41
		DDD	70	0^ii^	0^ii^
*Nicandra physaloides*	4	Mock-treated	12	0	0
(Solanaceae)		WT	33	16	48
		AAA	75	9	12
		DDD	63	13	21

^i^DNA-B variants were co-inoculated with AbMV DNA-A^WT^.

^ii^No systemic infection.

### Extraction of Total Nucleic Acids From Plants

100 mg leaf tissue from newly emerged sink leaves were collected and total nucleic acids (TNA) were extracted by a cetyltrimethylammonium bromide (CTAB)-based protocol according to Kleinow et al. (2009b). All plants (see 2.2, **Tables 1** and **2**) were harvested for TNA preparation and RCA-RFLP testing at 21 and 45 days post-inoculation (2.4). Samples from the independent experiments (see 2.2, **Tables 1** and **2**) from solanaceous hosts, for which a systemic infection with the respective AbMV variant was confirmed, were subjected to semi-quantitative Southern blot analysis at the time points indicated.

### RCA-RFLP Analysis and Direct Sequencing of RCA Products

AbMV circular DNA molecules in TNA samples (2.3) served as templates for RCA *via* a TempliPhi DNA amplification kit (GE Healthcare/Amersham Biosciences, Uppsala, Sweden) as described previously (Kleinow et al., 2009b; Jeske, 2018). The presence of the desired AbMV variants in the products was confirmed by RFLP analysis using MP mutant-specific restriction enzymes (*Alw *NI, *Blp *I or *Pvu *II) combined with *Bam *HI (**Figure 1**)(Kleinow et al., 2009b). Additionally, the integrity of each DNA-B encoded MP version was verified by direct sequencing of the RCA products (Schubert et al., 2007).

### Semi-Quantitative Southern Blot Analysis of the vDNA Content

TNA (1 µg) extracted from test plants (2.3) were subjected to semi-quantitative Southern blot analysis with DNA-A- and DNA-B-specific DNA probes. Sample separation on an agarose gel, blotting onto nylon membranes, hybridization, chemiluminescent detection of the digoxigenin (DIG)-labelled probes, quantification, and stripping of membranes for re-probing were carried out as detailed earlier (Kleinow et al., 2009b). Numbers of independent experiments are stated in 2.2 and 2.3.

### Detection of Protein Interactions in Yeast Two-Hybrid Assays

Yeast two-hybrid analysis using the GAL4-based Matchmaker System (BD Biosciences/Clontech, Palo Alto, CA, USA) were performed as specified (Kleinow et al., 2009a; Krenz et al., 2010). Briefly, yeast two-hybrid expression constructs for GAD::HA fusion proteins (pcACT2 or pACT2 Leu^+^) were transformed into *Saccharomyces cerevisiae* strain Y190 (MATa) and those for GBD fusion proteins (pAS2-1, Trp^+^) into strain Y187 (MATα) using a LiCl-based protocol. Transformants were selected on synthetic defined (SD) minimal medium plates lacking either Leu or Trp for three to five days at 30°C. Both plasmids were then combined in yeast cells by mating according to the Matchmaker system manual (BD Biosciences/Clontech). Protein interaction was monitored by activation of the reporter genes encoding yeast imidazoleglycerolphosphate dehydratase (HIS3) and bacterial β-galactosidase (lacZ). A colony-lift filter assay was used to test yeast cells grown on SD medium plates depleted for both Leu & Trp (selection for both plasmids) and additionally on plates lacking Leu, Trp & His supplemented with 50 mM 3-amino-triazole (3-AT; His3 activation test, Matchmaker system manual) for β-galactosidase activity. The presence of GAD::HA and GBD fusion proteins was confirmed by Western blot analysis (2.9; **Figure 6**). In total three independent experiments were performed. Each of those included as a positive control the construct GBD::At1g79160 (*Arabidopsis thaliana*), which activates the reporter genes autonomously, and as a negative control GAD::HA and GBD fusions of At3g27500 (*A. thaliana*) (Kleinow, 2000). In total, three independent mating assays and subsequent colony-lift filter tests were carried out.

### Transient Expression of AbMV MP in *N. benthamiana*

For transient expression, binary FRET constructs (2.1) were agro-infiltrated at an agrobacterium concentration of OD_600nm_ 0.25 into fully expanded third to fourth leaves of three-weeks old *N. benthamiana* plants according to Schütze et al. (2009). To optimize protein yield, a 35S promoter-driven expression construct for the p19 silencing suppressor of Cymbidium ringspot virus (Silhavy et al., 2002) was co-infiltrated (same agrobacteria concentration as for FRET constructs). Fluorescence microscopy and FRET analysis was performed at three days post agrobacteria infiltration (dpai). Presence of mVenus and mTurquoise2 fusion proteins was confirmed by Western blot analysis and in-gel fluorescence detection (2.9; **Supplementary Figure S1**).

### Live-Cell Imaging to Monitor Subcellular Localization and Detection of Protein Interactions *In Planta* by Acceptor Photobleaching-FRET Analysis

For microscopic investigations, leaf discs (diameter 1 cm) were collected from agro-infiltrated *N. benthamiana* plants and infiltrated with perfluorodecalin (PFD) for enhanced optical resolution (Littlejohn et al., 2010). Samples were mounted onto glass slides and sealed with cover slips. High resolution fluorescence microscopy was carried out with an inverted confocal Zeiss AxioObserver CSU-X1 Spinning Disk Unit with UGA-42-Firefly point scanning device (ZEISS Oberkochen, Germany; Rota Yokogawa GmbH & Co. KG, Wehr, Germany; Rapp OptoElectronic GmbH, Wedel, Germany). Collection of fluorescence intensities for FRET analysis and inspection of the subcellular localization of mTurquoise2- and mVenus-tagged proteins was done with the following microscope settings: Fluorescence of the donor mTurquoise2 was obtained with a 445 nm diode excitation laser combined with 485/30 nm emission filter. The acceptor mVenus was excited with a 514 nm laser, and fluorescence recorded upon use of a 562/45 nm emission filter. Images were acquired using an Axiocam 503 mono (CCD) camera in 2x2 binning mode. Bleaching was performed for an area nearly equivalent to the field of view (excluding only a small outer edge, **Figure 7A**), using an accessory high-intensity 514 nm bleaching laser device, purpose-built for specimen photomanipulation (UGA-42-Firefly point scanning device). 60% laser intensity was applied for ca. 50 s to ensure uniform mVenus bleaching. This bleaching set-up was applied in all experiments to minimize fluorescence recovery within the analyzed area. Fluorescence intensities of mVenus were recorded before and after the bleaching treatment for follow-up evaluation of bleaching efficiencies. A representative example of fluorescence micrographs imaged before and after specific bleaching of mVenus (acceptor) is shown in **Figure 7A**. To quantify FRET signals, the intensity of donor (mTurquoise2) fluorescence was evaluated prior to, and after acceptor photobleaching (**Figure 7A**). Recording of the overall FRET data set for mTurquoise2 and mVenus fluorescence before and after bleaching took around 55 sec per cell. To discrimnate occasional randomly occurring FRET signals from those caused by specific MP-MP interaction, a reference control for background FRET, which may result, e.g., from random accumulation of test proteins in certain membrane domains and thereby close proximity to each other, was applied. Such random FRET events were monitored by the combination of a plasma membrane-targeted soluble N-ethylmaleimide-sensitive-factor attachment receptor (SNARE) protein (*A. thaliana* SYP122; mVenus::SYP122) (Uemura et al., 2004) with mTurquoise::MP^WT^ (Happle et al., submitted). Both were expressed in *N. benthamiana* and analyzed in the same set-up. Only those FRET data sets of MP-MP tests were considered as positive that were significantly different from this control data set (statistical analysis by non-parametric Kruskal-Wallis test followed by Dunn’s multiple comparison of ranks). By applying this stringent baseline, the risk of false-positive results was minimized. The previously confirmed self-interaction of MP^WT^ (Krenz et al., 2010) served as a positive control. For each test combination, leaves of three plants were infiltrated per experiment, and at minimum three independent experiments conducted. For each test combination, ten cells were analyzed from every experiment, resulting in a data set of at least 90 cells/images for subsequent evaluation. For each of these cells/images, fluorescence intensities of donor and acceptor were measured in ten squared regions of interest (ROIs; size each 3.1623 × 3.1623 µm [= 10 µm^2^]; example shown in **Figure 7B**), along the plasma membrane/cell periphery, at identical positions and within the central part of the area used for acceptor bleaching, in images captured a second before and a second after photobleaching using Fiji (Image J) (Schindelin et al., 2012). Mean fluorescence intensities (F) after background subtraction, and effectiveness of bleaching and FRET efficiencies (FRET_eff_) were calculated using MS Excel (V 16.34) and Graphpad Prism (V8) for Mac. Data of cells with less than 90% bleach efficiency were excluded from the following evaluation. FRET_eff_ was ascertained as follows: FRET_eff_ = 1- (F^mTurquoise before bleaching^)/F^mTurquoise post bleaching^). All FRET_eff_ values obtained for each test combination were plotted and the median values with quartiles are determined (**Figure 7C**). The complete data sets collected in the absence and presence of AbMV infection were compared and statistically evaluated using Mann-Whitney tests (Mann and Whitney, 1947).

### Western Blot Analysis of Yeast and Plant Total Protein Extracts, and In-Gel Fluorescence Assays

Total proteins from yeast cells from the mating assays described in 2.6 were extracted by a urea/SDS-based method (Printen and Sprague, 1994). Yeast cells were grown in 5 ml Yeast Extract–Peptone–Dextrose (YPD; Matchmaker system manual) medium at 30°C up to a minimal OD_600nm_ of 0.7. Cells were harvested by centrifugation (6 min, 1,490 x*g*, RT). Per OD_600nm_ unit of cells, 13.5 µl extraction buffer (8 M urea, 5% (w/v) SDS, 40 mM Tris-HCl pH 6.8, 0.1 mM EDTA, 0.4 mg/ml bromophenol blue, 1% β-mercaptoethanol; pre-warmed to 70°C) and an 80 µl-volume glass beads (200 nm, acid-cleaned) were added. The suspension was vortexed for 1 min, incubated at 70°C for 10 min and at 40°C for 30 min. The suspension was cleared by centrifugation (5 min, 12,000 x*g*, RT), the supernatant transferred to a fresh tube and either subjected to SDS-PAGE directly or stored at -80°C.

Total proteins from plant samples described in 2.7 were extracted as follows: 100 mg leaf material was homogenized at 4°C in 200 µl extraction buffer (10 mM KCl, 100 mM Tris-HCl pH 8.0, 5 mM MgCl_2_, 400 mM sucrose, 10% (v/v) glycerol, 0.1% β-mercaptoethanol) using a plastic pestle and the homogenate cleared by centrifugation (5 min, 12,000 x*g*, RT). Prior to electrophoresis, 4x SDS-PAGE loading buffer (8% SDS, 400 mM Tris-HCl pH 8.8, 100 mM DTT, 40% glycerol and 0.064% bromophenol blue) was added to all samples.

After heating to 40°C for 30 min, proteins of yeast or plant extracts were separated on a 12.5% SDS-PAGE and transferred onto nitrocellulose membranes by semi-dry blotting according to Kleinow et al. (2008). Successful transfer was monitored by Ponceau-S staining. Gels containing FRET expression construct samples were analyzed for mVenus- and mTurquoise2-tagged proteins prior to blotting by in-gel fluorescence detection (mTurquoise2: excitation 440 nm/emission 535 nm and mVenus: excitation 480 nm/emission 535 nm; Fusion fx7edge imaging system, Vilber Lourmat, Eberhardzell, Germany; **Supplementary Figure S1**). Western blot analysis was carried out as described (Kleinow et al., 2008) with minor modifications, using a blocking solution with 2.5% bovine serum albumin (BSA, fraction V) in Tris-buffered saline buffer (TBS, 20 mM Tris-HCl, 138 mM NaCl). Yeast-expressed proteins fused to GBD were detected with a monoclonal anti-GBD antibody (RK5C1) from mouse (1:7,000; Santa Cruz Biotechnology, Dallas, TX, USA) and an anti-mouse horseradish peroxidase conjugated antibody from goat (1:10,000; Rockland, Gilbertsville, PA, USA). For immunodetection of GAD::HA fusion proteins, a rat anti-hemagglutinin (HA) high affinity antibody (3F10) (1:2,000; Roche, Mannheim, Germany) was combined with a goat anti-rat secondary IgG coupled to horseradish peroxidase (1:10,000; Sigma, Taufkirchen, Germany). Monoclonal anti-GFP antibodies (mixture of two antibodies 7.1 and 13.1) from mouse (1:1,000; Roche) recognizing both WT and mutant forms of GFP such as mVenus and mTurquoise2, and an anti-mouse horseradish peroxidase conjugated antibody from goat (1:10,000; Rockland) was used for immunodetection of plant-derived FRET test proteins. Detection of all target proteins was performed *via* an enhanced chemiluminescence method (Luminata™ Crescendo, Merck EMD Millipore, Schwalbach, Germany; Fusion fx7edge imaging system, Vilber Lourmat).

## Results

### Functional Analysis of DNA-B Variants Encoding Triple Phosphorylation Site Mutations (T221, S223, and S250) of MP *In Planta*

To analyze the functional role of AbMV MP phosphorylation *in planta* in closer detail, two novel infectious DNA-B constructs were generated that encode MPs with triple amino acid substitutions for T221, S223 and S250 against either aspartate (MP^DDD^; “constant-on of charge”) or alanine (MP^AAA^; “constant-off of charge”) in all three positions. There are several examples available in the literature that describe a loss of infectivity for specific host plant species due to clonally selected geminiviral DNA molecules (for a more detailed discussion see Briddon et al., 1992; Briddon et al., 1998; Grigoras et al., 2009; Jeske et al., 2010; Richter et al., 2016a; Richter et al., 2016b; Jeske, 2018). Thus, RCA-based inocula were applied to circumvent such potential limitations of single virus DNA clones, and to preserve the quasi-species nature of the vDNA pool in the experiments. An *in vitro* RCA-based cloning strategy using the previously established mutated DNA-Bs as starting material was applied in order to maintain the vDNA population (**Figure 1**; 2.1) (Kleinow et al., 2009b). According to the preceding experiments described in Kleinow et al. (2009b), *N. benthamiana* plants were biolistically inoculated with RCA products of AbMV DNA-A plus DNA-B, either WT or encoding one of the two triple phosphorylation site MP mutants. Combinations comprising DNA-A and DNA-Bs expressing the formerly investigated single and double phosphorylation site mutants of MP (Kleinow et al., 2009b) and mock-treated plants were processed in parallel as references or healthy controls, respectively. All AbMV test combinations resulted in a systemic infection with the respective variant, as confirmed by RFLP analyses using a mixture of *Bam *HI (cuts twice in DNA-B) and either of the mutant-specific restriction enzymes, as well as by direct sequencing of RCA products amplified from TNA from newly emerging sink leaves (example in **Figure 1**; **Table 1**; 2.2–2.4).

To test the stability of the newly generated DNA-B-MP^DDD^ or -MP^AAA^ versions *in planta*, the virus pools of three individual, primary-inoculated plants per mutant type were amplified by RCA and the products from each of those plants separately passaged *via* biolistic inoculation to another set of four plants (**Table 1**). These secondary-inoculated plants were evaluated for the presence of the correct mutations by RCA-RFLP and sequencing as well, and the results confirmed the maintenance of the respective mutant DNA-B forms. Eight primary inoculation experiments (with in total 42 plants) and one passaging test (with in total 12 plants) per DNA-B encoding an MP triple mutant were performed (**Table 1**).

**Figure 2:**
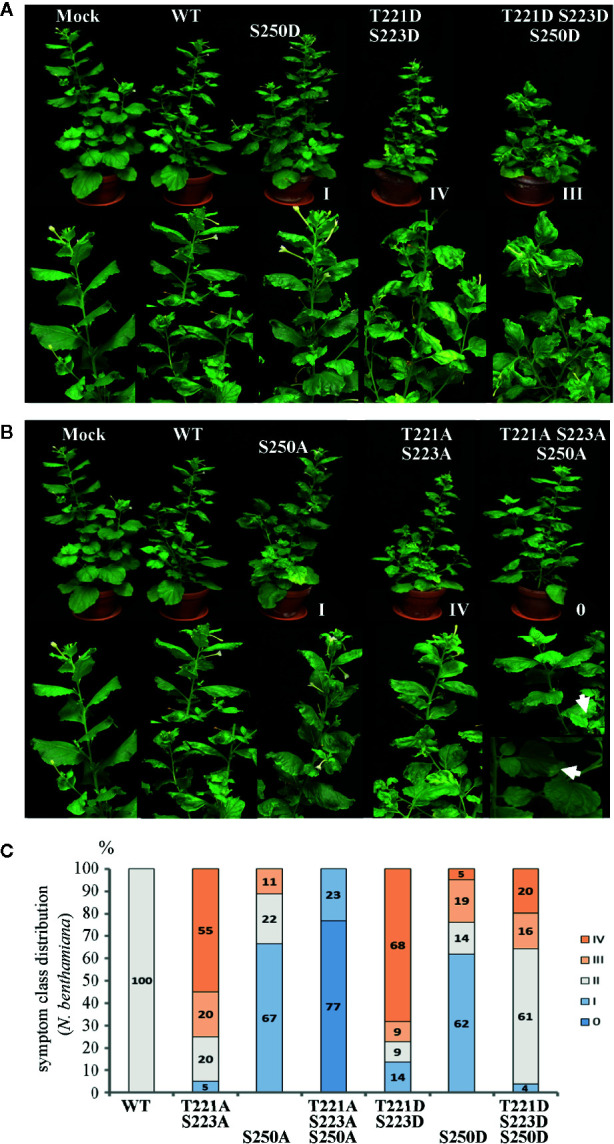
Alterations in AbMV symptom development triggered by MP with either single, dual, or triple aspartate (DDD) or single up to triple alanine (AAA) replacement for amino acid positions T221, S223 and S250. *N. benthamiana* plants were inoculated with AbMV DNA-A plus DNA-B containing a *MP* gene of either wild-type or the distinct mutated versions indicated. The previously described single and double phosphorylation site mutants of MP (Kleinow et al., 2009b) and mock-treated plants served as reference for the novel triple mutation containing MP. The plant classification according to symptom severity (**Table 1**) is indicated. **(A)** Appearance of plants (48 dpi) systemically infected with the indicated phosphorylation-mimicking aspartate replacement mutants, and **(B)** with analogous alanine exchanges. A close-up is shown for symptoms below each plant. Note the nearly symptomless phenotype of MP^AAA^ combination. Small leaf areas displaying yellowing or mild crinkling are marked with an arrow. **(C)** Summary of symptom class distribution for nine independent experiments.

The symptoms of *N. benthamiana*, proven to be infected with the respective AbMV mutant (2.3–2.4), were scored in comparison to mock-inoculated and AbMV^WT^-infected ones. With respect to the evaluation criteria - severity of stunting; extent of yellow-green leaf mosaic; and leaf deformations - , plants were grouped into five symptom categories (**Table 1**, **Figure 2**). Experiments with primary- and secondary-inoculated plants were combined, as there was no significant difference between them in symptom development and infection rates (**Table 1**, **Figure 2**). Overall, the analyses by RCA-RFLP, direct sequencing of RCA products, evaluation of infectivity, and symptom development did not reveal any instability of the mutated DNA-B versions and showed equivalent reactions of the primary- and secondary-inoculated plants, suggesting a controlled variability within the samples and a dominant influence of the respective MP versions. All tests and the reproducible outcomes indicate uniformity and repeatability in the experimental set-up using RCA products as inocula.

Representative plants are shown in **Figure 2**. As expected from earlier experiments, AbMV encoding MP^DD^ and MP^AA^ double mutants for T221 and S223 exhibited significantly more severe symptoms in the majority of plants (77 and 75%, respectively, in category III and IV), whereas the ones for S250 developed predominantly milder phenotypes (62 and 67% in category I) (**Table 1**, **Figure 2**) (Kleinow et al., 2009b). Both MP triple mutants induced a divergent, amino acid substitution-specific symptom development in contrast to the amino acid position-specific effects observed with the single and double mutants. The AbMV^DDD^ symptoms were similar to those of the WT virus in the majority of plants (61% in category II), enhanced in 36% (category III and IV) and attenuated in around 4% (**Table 1**, **Figure 2**). On the contrary, AbMV^AAA^ induced extremely mild symptoms (100% category I and 0) (**Table 1**, **Figure 2**). Young leaves were symptomless and older ones exhibited some yellow spots or small areas of leaf crinkling only occasionally (example in **Figure 2**, close-up).

The steady-state vDNA content of systemically infected sink leaves was determined by semi-quantitative Southern blot analysis to monitor the impact of three simultaneously altered phosphorylation sites on MP functions and AbMV spread. *N. benthamiana* plants verified to be systemically infected with the correct combination of DNA-A and the respective DNA-B variant (MP^DDD^ or MP^AAA^) by RCA-RFLP and sequencing were assayed. AbMV^WT^-infected plants served as a reference. Samples from 14, 26, and 49 dpi were analyzed for time-dependent changes in vDNA accumulation.

**Figure 3:**
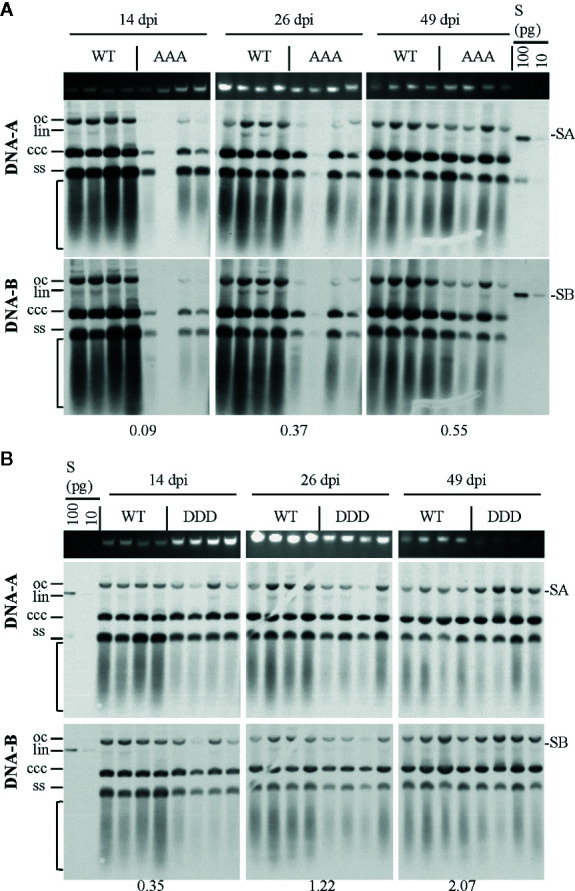
Effect of non-phosphorylation and phospho-charge-mimic MP mutants on AbMV DNA accumulation in *N. benthamiana*. Total nucleic acids were extracted at indicated days post inoculation (dpi) from plants systemically infected with AbMV DNA-A plus one of the three DNA-B variants coding for MP^WT^ **(A, B)**, MP^AAA^ **(A)** or MP^DDD^ **(B)**. Four samples for each combination were separated on an agarose gel in the presence of ethidium bromide, and the area harboring plant genomic DNA served as a loading control (**A**, **B**; upper panel). Semi-quantitative Southern blot analyses were carried out with a DNA-A-specific probe (**A**, **B**; middle panel) and, after stripping and re-hybridization, with a DNA-B-specific probe (**A**, **B**; lower panel). Different vDNA forms are indicated (oc: open circular, lin: linear, ccc: covalently closed-circular and ss: single-stranded). The area indicative of replication by-products is labelled by a bracket. The intensity of hybridization signals was normalized to the amount of DNA loaded. For each sample set, the mean of four typical samples was calculated, and a factor determined for the vDNA difference between WT and triple mutants per time point (displayed below). S: hybridization standards (left to right 100 and 10 pg of standard A [SA]: PCR-amplified DNA A-encoded ORF *AC1* or standard B [SB]: PCR-amplified DNA B lacking sequence of common region). Southern blot analysis was performed for three to four samples per independent experiment with *N. benthamiana* (**Tables 1** and **2**).

Systemic infection with AbMV expressing MP^AAA^ resulted in a transiently delayed accumulation of vDNA (**Figure 3A**). The vDNA content was substantially reduced at 14 dpi, but had increased to approximately half of that of AbMV^WT^ at 49 dpi. The AbMV^DDD^ mutant contained slightly reduced vDNA levels at 14 dpi, but accumulated vDNA to higher levels than AbMV^WT^ at later time points (**Figure 3B**). A difference between the time courses of DNA-A and DNA-B accumulation for MP^AAA^ and MP^DDD^ expressing AbMV variants was not detected (**Figure 3**), in contrast to previous results (Kleinow et al., 2009b).

Overall, the triple replacements by alanine interfered with pathogenicity and vDNA accumulation, whereas that by aspartate showed a weaker influence on the infection in *N. benthamiana* plants.

### The Influence on Symptom Development of MP^AAA^ and MP^DDD^ Depends on the Host Plant Species

For MP^AAA^ and MP^DDD^ variants of AbMV tests were carried out to examine whether these mutations would differentially affect the infection of hosts other than *N. benthamiana* and thus could alter the viral host range. Three plant species susceptible for AbMV^WT^ in the families Solanaceae (*Datura stramonium* and *Nicandra physaloides*) and Malvaceae (*Malva parviflora*) (Jeske, 2000; Wege et al., 2000) were chosen. Seedlings in the two-to-three-leaf stage were biolistically inoculated with RCA products of viral AbMV DNAs-A and each MP^AAA^, MP^DDD^, or MP^WT^ encoding DNAs-B. Mock-treated *N. benthamiana* plants were processed in parallel. The various hosts were inspected for systemic AbMV infection by monitoring symptoms and by testing vDNA from newly emerging sink leaves by RCA-RFLP and Southern blot hybridizations.

AbMV^WT^ infected all hosts with different efficiencies (**Table 2**). For each of the MP mutants the results were plant species specific: For *M. parviflora*, AbMV^AAA^ was not infectious at all, whereas AbMV^DDD^ achieved lower infection rates than AbMV^WT^ (average infection rates 22% versus 55%; **Table 2**). For *D. stramonium,* AbMV^DDD^ was not infectious, and AbMV^AAA^ was similar to AbMV^WT^ (**Table 2**). For *N. physaloides*, both AbMV^AAA^ and AbMV^DDD^ were systemically infectious, although with reduced infection rates (12 or 21%, respectively).

**Figure 4:**
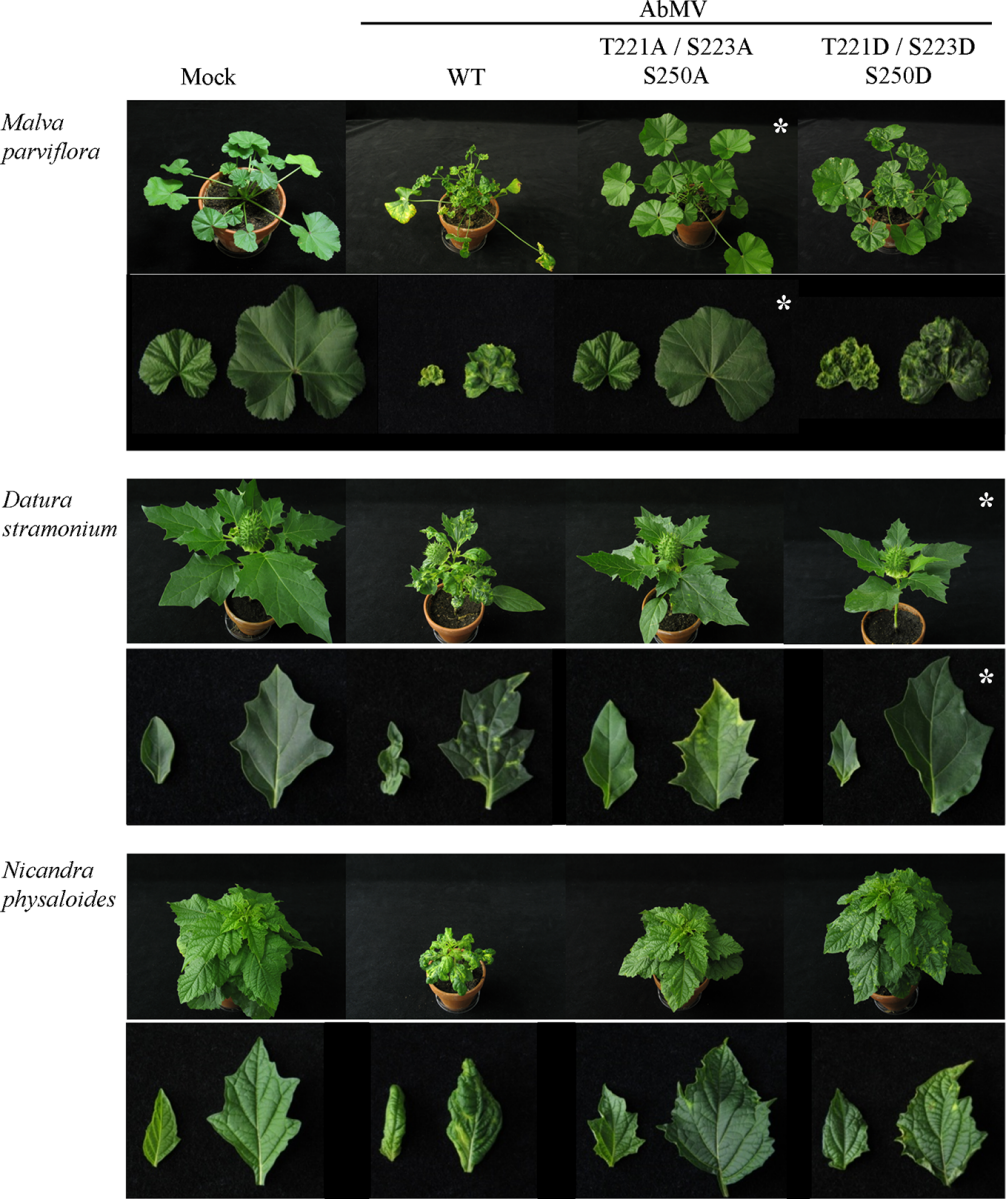
Impact of triple phosphorylation site mutants of MP on AbMV symptom development in host plants other than *N. benthamiana*. *Malva parviflora*, *Datura stramonium*, and *Nicandra physaloides* were inoculated with DNA-A plus DNA-B encoding MP^WT^, MP^AAA^, or MP^DDD^ in at minimum four independent experiments (**Table 2**). Mock treatments served as a negative control. Each panel shows the whole plant (35 dpi) and directly below a close-up (right young and left old leaf). Non-infectious virus-host combinations are labeled by an asterisk (compare ; AAA & *M. parviflora*, DDD & *D. stramonium*). Note that all combinations comprising a triple phosphorylation site mutant version of MP exhibit attenuated symptoms in comparison to AbMV^WT^.

All three plant species developed stronger symptoms upon systemic AbMV^WT^ infection than for *N. benthamiana.* Symptoms comprised more severe stunting, leaf deformations and yellow-green leaf mosaic (**Figure 4**). In contrast, both MP triple mutants caused milder symptoms in the three hosts, if infected. Young sink leaves were nearly symptomless, and older source leaves showed minor wrinkling and small yellow spots rather than a yellow-green mosaic for *D. stramonium* and *N. physaloides* (**Figure 4**).

**Figure 5:**
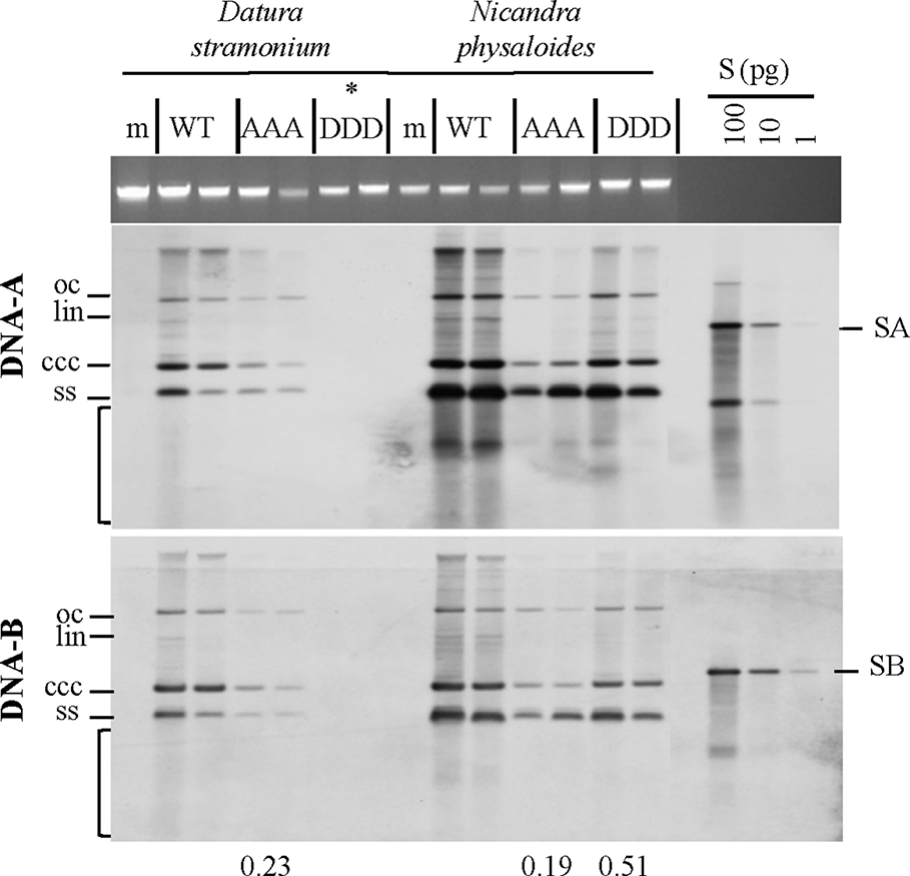
Triple phosphorylation site mutants of MP influence vDNA accumulation in solanaceous host plants. Semi-quantitative Southern blot analyses of total nucleic acids extracted from *D. stramonium* and *N. physaloides* at 44 dpi were carried out as for **Figure 3**. Two plant samples for each combination are shown (m: mock-treated control; WT: AbMV^WT^; AAA: AbMV^AAA^; DDD: AbMV^DDD^). Note that AbMV^DDD^ is non-infectious in *D. stramonium* (asterisks; compare **Table 2**). Different vDNA forms are indicated (oc: open circular, lin: linear, ccc: covalently closed-circular, ss: single-stranded and bracket: area indicative of replication by-products). Data normalization and calculation of factor for the vDNA difference between WT and triple mutants per plant species, as explained in **Figure 3** (displayed below). SA: DNA-A-specific hybridization standard; SB: DNA-B-specific hybridization standard (left to right 100 and 10 pg). Southern blot analysis was done for two to three samples out of each independent experiment (summarized in **Table 2**).

Southern blot hybridizations were performed for samples from plants infected with the AbMV versions, as verified by RCA RFLP and sequencing, to test for vDNA accumulation. In comparison to AbMV^WT^, in all AbMV^AAA^-infected *N. physaloides* and *D. stramonium* the vDNA content was markedly reduced at 44 dpi (**Figure 5**), whereas this effect was less prominent with MP^DDD^ expressing AbMV. No significant difference in those trends was found between DNA-A or DNA-B titers.

In summary, the AbMV triple mutants exerted complex differential effects on hosts, regarding symptom development and vDNA accumulation. Both mutants have lost infectivity for a host susceptible for AbMV^WT^, suggesting a primary role of the three phosphorylation sites in fine-tuning virus-host interplay and thereby efficiency or even ability of systemic host invasion.

### Triple Mutations Influence the Self-Interaction of MP

**Figure 6:**
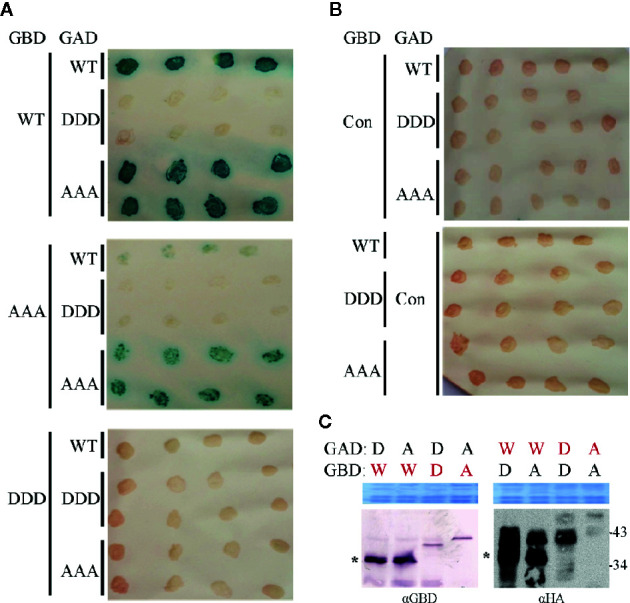
Influence of triple phosphorylation site mutations on MP self-interaction in a yeast-two hybrid system. **(A)** Qualitative colony-lift LacZ filter assay to monitor interactions of GAD::HA and GBD fusions of MP oligomerization domains (MP-CD) between either WT or mutagenized (DDD or AAA) variants. The confirmed interaction of the WT protein served as a positive control. **(B)** Test combinations with GAD::HA and GBD fusions of a Cys/His-rich C1 domain family protein from *A. thaliana* (At3g27500; Con) were tested in parallel as a negative non-interacting control. **(C)** Western blot analysis to assay the presence of the respective GAD::HA and GBD fusion test proteins (depicted in red letters). Expected molecular mass for MP-CD variants fused to GBD is 36.7 kDa and to GAD::HA is 38.7 kDa. W: MP-CD^WT^; D: MP-CD^DDD^; A: MP-CD^AAA^. Asterisk: band position expected in relation to right: (apparent) kDa deduced from molecular weight marker. An area of the Coomassie-stained SDS-PAGE is shown as a loading control. In total three independent experiments were carried out.

Both MP mutant types were tested in yeast two-hybrid (Y2H) assays in *S. cerevisiae* cells. To provide the assay functionality, truncated C-terminal domains (MP-CD) were used lacking the membrane anchor domain (Zhang et al., 2002), since full-length MP fusion proteins target to the cell periphery preventing Y2H interactions (Frischmuth et al., 2004). Fusion constructs of all MP-CD variants with either GBD or GAD::HA were tested against each other in all possible combinations. The known self-interaction of MP-CD^WT^ served as positive control, and its combination with an unrelated protein from *A. thaliana* (Cys/His-rich C1 domain family protein, At3g27500) as negative control. Test constructs were combined using single transformations and a mating assay. Yeast cells were tested for reporter gene activation indicative of protein interaction by monitoring histidine prototrophy and ß-galactosidase activity. MP-CD^AAA^ interacted in all combinations with itself or with MP-CD^WT^. In contrast, no protein interaction was detectable for the DDD variant in any combination (**Figure 6A**). An interaction with the control construct was not detectable likewise (**Figure 6B**, Con). The expression of the GAD::HA- and GBD-fused proteins was confirmed by Western blot analysis (**Figure 6C**). In accordance with previous results (Kleinow et al., 2008; Kleinow et al., 2009b), MP-CD^WT^ showed signals at the expected apparent molecular mass and retarded extra bands (**Figure 6C** right panel). Interestingly, for both mutated MPs only bands with reduced mobility in comparison to the MP-CD^WT^ appeared.

The self-interaction of full-length MP was further tested in living plant cells, using an acceptor photobleaching bleaching FRET detection approach with the fluorophore pair of acceptor mVenus and donor mTurquoise2 (Blatt and Grefen, 2014; Hecker et al., 2015). FRET efficiency measurement is a highly stringent and specific method to assay if two proteins fused to the fluorescent reporters are in close proximity in a protein complex with a distance less than 10 nm (Hecker et al., 2015). When proteins interact only temporally, FRET vanishes upon protein partner dissociation. This additional advantage of FRET allows detecting temporal changes in MP self-interaction *in planta*. The BiFC technique, which had been used previously to confirm MP^WT^-MP^WT^ interaction in *N. benthamiana* (Krenz et al., 2010), is limited in this respect because complexes of proteins fused to yellow fluorescent protein (YFP) domains may undergo stabilization upon reconstitution of the YFP ß-barrel structure (Hu et al., 2002; Magliery et al., 2005).

**Figure 7:**
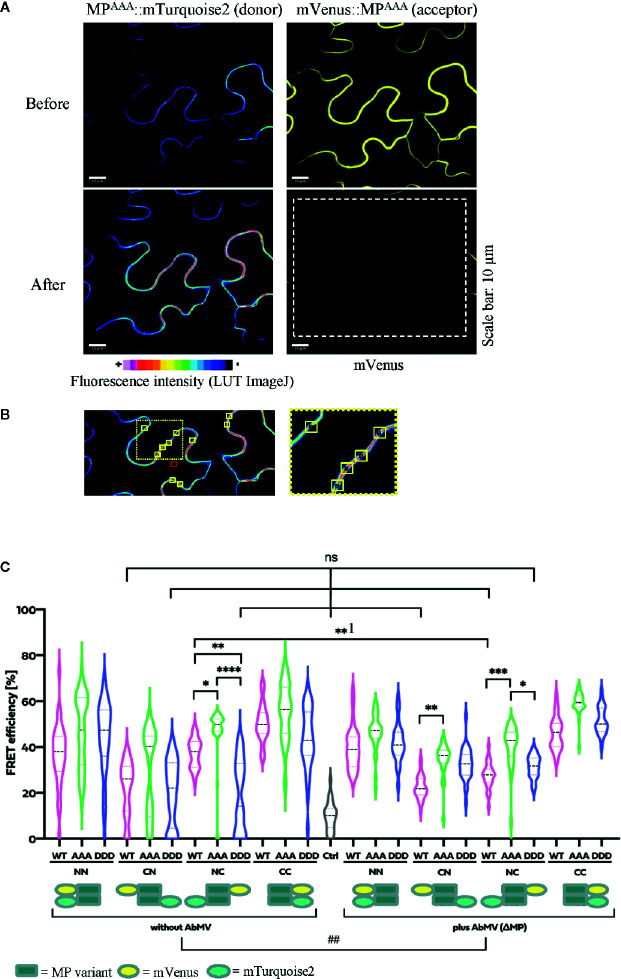
Functional relevance of MP phosphorylation site triple mutants for self-interaction in healthy or AbMV-infected *N. benthamiana*. Protein interaction was assayed by acceptor photobleaching-FRET using the acceptor mVenus and donor mTurquoise2. All four possible combinations of C- and N-terminal fusion to full-length MPs (WT: MP^WT^; DDD: MP^DDD^; AAA: MP^AAA^; see schematic representation) were transiently expressed after agro-infiltration in the absence or presence of AbMV (ΔMP gene) (Evans and Jeske, 1993) infection in leaf epidermal tissues. The combination of a plasma membrane-targeted SNARE protein (*Arabidopsis* SYP122; mVenus::SYP122) (Uemura et al., 2004) with mTurquoise2::MP^WT^ served as a negative control and all combinations of MP^WT^ (Krenz et al., 2010) as a positive control. High resolution spinning disk confocal microscopy was done at three dpai. **(A)** Representative example of fluorescence micrographs (combination MP^AAA^::mTurquoise2 & mVenus::MP^AAA^) imaged before and after specific bleaching of mVenus (acceptor; bleaching area marked by a dashed square). **(B)** Ten squared regions of interest (ROIs; size 10 µm^2^ each, yellow squares) along the plasma membrane were selected and analyzed per cell. An example is shown for a donor fluorescence micrograph after bleaching (area of close-up to the right indicated by a dotted square). Effectiveness of bleaching and FRET efficiencies were calculated as described in 2.8. Note the increased signal for the donor mTurquoise2 after bleaching the acceptor mVenus. For better visualization of fluorescence intensities, mTurquoise2 is displayed with ImageJ’s lookup table (LUT) black to white in 16 colors. Scale bar = 10 µm; images are single optical planes. **(C)** Intensities of acceptor and donor fluorescence were analyzed prior to and after acceptor photobleaching, and the resulting FRET efficiencies were calculated. For each sample, all data points of at minimum three independent experiments are presented as violin plot to better visualize their distribution, and median values with quartiles are depicted. Sample pairs with significant difference are labelled by asterisks: Kruskal-Wallis Test: P value ****<0.0001, ***0.0002, **0.001, *0.01; comparison of only healthy versus AbMV-infected: Mann-Whitney Test; ^##^P value 0.005; NS, no significant difference.

The two-in-one FRET binary vector system for the simultaneous expression of two potential interaction partners from a single T-DNA (Hecker et al., 2015) was applied to analyze the fluorescent MP fusion proteins at comparable levels. MP^DDD^ and MP^AAA^ were investigated in comparison to MP^WT^ fusion proteins. To reveal a putative impact of the position and sequence of the fluorescence protein tags on MP-MP interactions, all four possible orientations were generated and assayed for every MP variant: mVenus (acceptor) and mTurquoise2 (donor) tags, respectively, either both located N-terminally (NN) or C-terminally (CC), and additionally N-terminally mVenus plus C-terminally mTurquoise2 (CN) and vice versa (NC) (**Figure 7**, for details see schematic representation). The four FRET constructs for each MP type were transiently and simultaneously expressed after agro-infiltration into epidermal tissues of *N. benthamiana* leaves. To examine if other viral proteins, the vDNA, or AbMV-triggered changes in the cellular environment affected MP-MP interactions, the experiments were done in the absence and in the presence of an AbMV infection. AbMV was inoculated locally by co-infiltration of plasmids delivering infectious DNA-A and a mutant DNA-B with abolished MP^WT^ expression (DNA-BΔMP) (Evans and Jeske, 1993). In order to detect appearance of non-specific FRET, potentially resulting from random accumulation of test proteins in certain membrane domains and thereby close proximity, an additional negative control was included: the plasma membrane-targeted SNARE protein (*Arabidopsis* SYP122; mVenus::SYP122) (Uemura et al., 2004) combined with mTurquoise2::MP^WT^. MP^WT^ self-interaction (Krenz et al., 2010) was included as a positive control in all layouts tested. High resolution fluorescence microscopy and FRET acceptor photobleaching experiments were performed by imaging samples, before and after photobleaching by a high energy laser beam (example shown in **Figure 7A**). Calculation of FRET efficiencies after background subtraction was done as described in 2.8.

All test layouts showed FRET (NN, CN, NC, and CC, **Figure 7B**) with and without AbMV, indicating that all MP variants were capable for self-interaction. Full-length MP^DDD^ thus differed in the result from that with the partial MP-CD^DDD^ in the yeast two-hybrid assay. Possibly the other MP domains, or plant adapter proteins, rescued the effect of the DDD mutation observed in the yeast system. In the context of an AbMV infection, the variability of FRET efficiencies within each combination was significantly reduced, suggesting a stabilization of the MP complex in the presence of the viral elements. Without AbMV, data for MP combinations with low FRET efficiencies, namely the CN (mVenus::MP^variants^/MP^variants^::mTurquoise2) and NC (MP^variants^::mVenus/mTurquoise2::MP^variants^) combinations were not normally distributed and showed FRET values in the range of the negative control. This suggests that in these layouts, MP-MP interactions were impaired, most likely by the opposite positions of the fluorescent protein tags that may influence protein folding or accessibility of domains. A significant difference in FRET efficiency of the same MP-MP combination in the absence or presence of AbMV was detected only for MP^WT^ in the layout NC (**^1^ label in **Figure 7B**; mean value 36% without vs. 28% with AbMV). Similarly, a significant but moderate difference between MP^WT^, MP^AAA^, and MP^DDD^ could be observed in a single layout only (NC with/without AbMV, mean FRET efficiencies: MP^AAA^: 50/42% without/with AbMV, i.e., significantly higher than that of MP^WT^: (36/28% without/with AbMV) and MP^DDD^ (14/30% without/with AbMV). For most layouts, no significant difference in FRET efficiencies occurred (**Figure 7B**).

Expression and integrity of the respective test proteins *in planta* were confirmed by Western blot analysis of leaf tissue extracts using an antiGFP antibody reacting with both fusion partners (**Supplementary Figure S1**). Additionally, the presence of the fluorescent protein-tagged MPs in the samples was confirmed by direct in-gel fluorescence detection (**Supplementary Figure S1**). Both assays showed expression of the MP fusion proteins and even allowed discrimination of distinct constructs, although their calculated molecular mass is nearly identical (C-terminally mTurquoise2 or mVenus 61.85 kDa; N-terminally mTurquoise2 or mVenus 61.82 kDa). All MP fusion variants exhibited a clearly different electrophoretic velocity indicative of N- or C-terminal tagging, irrespective if fused to mVenus or mTurquoise2. The C-terminal fusion proteins migrated more slowly than the N-terminally fused ones. All tagged MP variants showed an anomalous migration by comparison to the molecular weight marker.

In order to investigate a potential impact of the phosphorylation sites on the subcellular localization of AbMV MP in living plant cells, high resolution spinning disk confocal microscopy of leaf areas containing fluorescence protein-tagged MP variants was carried out. MP^DDD^ and MP^AAA^ were analyzed in comparison to the WT protein. For this purpose, the four different FRET expression constructs for each MP type were transiently expressed in epidermal leaf tissues of *N. benthamiana* following agro-infiltration. Experiments were carried out without and with local AbMV infection.

**Figure 8:**
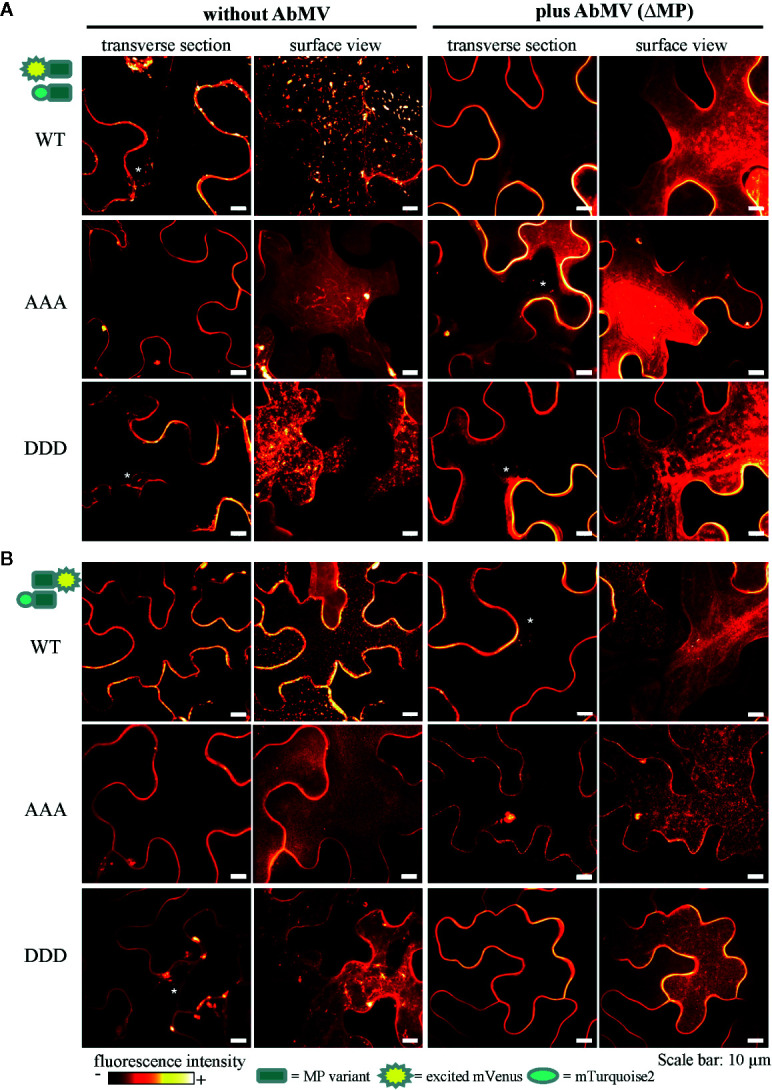
Subcellular localization of AbMV MP phosphorylation site triple mutants. *N. benthamiana* transiently expressing combinations of full-length MP FRET constructs and the negative control set-up (as in **Figure 7**) were analyzed by high resolution spinning disk confocal microscopy at three dpai. Images show the localization of mVenus-tagged test proteins **(A)** mVenus::MP^WT/AAA/DDD^ and **(B)** MP^WT/AAA/DDD^::mVenus, which was indistinguishable from that found with mTurquoise2 fusion proteins (not shown). Additionally, no difference between C- or N-terminal mVenus MP fusion proteins in combinations with either N- or C-terminally mTurquoise2-tagged MP variants was detected (for N- or C-terminally mVenus-tagged MPs combined with same MP type in C-terminal mTurquoise2 fusion, see **Supplement Figure 2**). Exemplary images of representative cells out of at minimum three independent experiments per treatment are shown in transverse section and surface view (displayed in a fluorescence intensity view). Nuclei are labeled by asterisks. Scale bars: 10 µm.

MP^WT^ showed the following subcellular distribution patterns: In cells devoid of AbMV co-infection, fluorescence signals for N- and C-terminally-tagged MP^WT^ localized homogeneously at the cellular periphery, most likely at the cytoplasmic face of the plasma membrane and at plasmodesmata (Happle et al., submitted; Aberle et al., 2002; Frischmuth et al., 2004; Kleinow et al., 2009c) (**Figure 8**; **Supplementary Figure S2**; exemplary fluorescence micrographs for mVenus). Fluorescent protein-tagged MP^WT^ accumulated around nuclei and clustered in small vesicles representing microsomes trafficking *via* the ER/actin network (Happle et al., submitted). The MP vesicles were highly mobile and traveled along the cell periphery and in close vicinity to the nuclear envelope within the cytoplasm. Small spotted structures detected for this fusion protein at the cell periphery exhibited Brownian molecular motion only, considerably different from the trajectories of the N-terminally tagged MP (**Figure 8**; **Supplementary Figure S2**; exemplary fluorescence micrographs for mVenus). As described in detail by Happle et al. (submitted), the proportions of cells with MP fusion proteins accumulating in vesicles, and cells with homogenous MP distribution at the cell periphery varied at three dpai. In the presence of AbMV, MP^WT^ localized predominantly at the cell periphery and formed fewer vesicles.

In all layouts investigated, the subcellular localization of both triple mutants was not significantly altered in comparison to that of MP^WT^. Exceptionally, a higher abundance of MP-containing vesicles occurred for MP^AAA^, and MP^DDD^- or MP^AAA^-containing filamentous structures at the cell periphery were occasionally detected in different layouts (examples **Figure 8A**, MP^AAA^; **Supplementary Figure S2A**, MP^DDD^).

## Discussion

Based on our former work that identified three phosphorylation sites in the C-terminal oligomerization domain of MP (T221, S223, and S250) (Kleinow et al., 2009b), this study has focused on triple mutants MPs. Site-directed mutagenesis of the *MP* gene within the infectious AbMV genome has generated a non-phosphorylation (“constant-off of charge”; AAA) or, alternatively, a phospho-charge-mimic (“constant-on of charge”, DDD) status for all three amino acid positions simultaneously. Upon systemic infection with AbMV^DDD^, about two thirds of the *N. benthamiana* plants developed symptoms similar to WT, and one third more severe symptoms. Time course experiments indicated changes in the steady-state vDNA content in comparison to AbMV^WT^, slightly reduced at early time points and elevated in later stages of infection. The vDNA content appeared to be independent of the symptom phenotype. In contrast, AbMV^AAA^ resulted in an extreme attenuation of symptoms up to nearly symptomless plants, and accordingly a time-delayed accumulation of vDNA. These results prove a vital role of the three phosphorylation sites located in the C-terminal part of AbMV MP for viral pathogenicity *in planta*. They are in line with other studies that identified the C-terminal part of begomoviral MPs as being important for symptom induction (von Arnim and Stanley, 1992; Ingham and Lazarowitz, 1993; Pascal et al., 1993; Duan et al., 1997; Hou et al., 2000; Saunders et al., 2001).

It is well established that PTMs and especially reversible phosphorylation are important elements in managing a multitude of processes in eukaryotic cells, e.g. altering protein function, stability or subcellular distribution. For MPs in the 30K family of various virus species, phosphorylation on serine and threonine residues was shown to be relevant for *in planta* functionality, including proteins of tobamoviruses (Kawakami et al., 1999; Waigmann et al., 2000; Karger et al., 2003; Kawakami et al., 2003; Trutnyeva et al., 2005; Fujiki et al., 2006), cucumoviruses (Matsushita et al., 2002; Lewsey et al., 2009; Nemes et al., 2017), bromoviruses (Akamatsu et al., 2007) and geminiviruses (Kleinow et al., 2009b). However, details of the regulatory events and reaction cascades triggered by changes of the phosphorylation patterns still need to be investigated.

To some extent, however, these findings for the novel triple phosphorylation site MP mutants contrast with data obtained in our former study (Kleinow et al., 2009b). While for the simultaneous triple changes, mutation-type dependent alterations in pathogenicity dominate the outcome, the previously analyzed single and double mutant versions revealed primarily amino acid position-specific effects on symptom development. On that former basis, we had hypothesized that an interference with phosphorylation-induced ubiquitination of proteins might result in amino acid position-specific effects in plants and change viral symptoms (for a more detailed discussion see Kleinow et al., 2009b). Recent data for the CP of the bipartite begomovirus African cassava mosaic virus support the idea of phosphorylation-mediated proteolysis as an important regulatory cornerstone: a differential CP phosphorylation in plants coinciding with a high turnover rate of the protein indicates its ubiquitin-dependent degradation by the proteasome (Hipp et al., 2019). An ubiquitination and subsequent degradation by the proteasome has also been observed for the CP of the monopartite begomovirus tomato yellow leaf curl virus (Gorovits et al., 2014; Gorovits et al., 2016).

Most interestingly, AbMV^AAA^ and AbMV^DDD^ changed the host ranges, with MP^AAA^ unable to infect *M. parviflora* and MP^DDD^ being non-infectious to *D. stramonium*. It can be speculated that site-selective phosphorylation in the MP oligomerization domain goes along with an adaptation of AbMV to specific plant species, as a prerequisite for successful infection and invasion. To the best of our knowledge, such phosphorylation-dependent host range modulation is shown for a MP of a plant DNA virus for the first time.

For some RNA viruses, however, host dependent phosphorylation of MPs and specific functional consequences e.g. on infectivity and host range, had been found before. Phospho-charge-mimic mutations of three C-terminally located phosphorylation sites in TMV MP affected its ability to gate plasmodesmata for intercellular trafficking in *Nicotiana tabacum*, but not in *N. benthamiana*. This unraveled a host dependency of TMV (genus *Tobamovirus*, family *Virgaviridae)* on specific phosphorylation patterns (Waigmann et al., 2000). For the movement-associated triple gene block protein 1 (TGB1p) of barley stripe mosaic virus (genus *Hordeivirus*, family *Virgaviridae*), two phosphorylation sites were identified (T395 and T401) that upon mutation (non-phosphorylation or phospho-charge-mimic) led to a change in host specificity (Hu et al., 2015). In TGBp3 of potato mop-top virus (genus *Pomovirus* within the *Virgaviridae*), a tyrosine phosphorylation site (Y120) was identified, the alanine substitution of which eliminated infectivity (Samuilova et al., 2013). All these data collectively underscore that MP phosphorylation/dephosphorylation dynamics may have a strong impact on the specific interplay between a virus and the inoculated plant species. The data obtained for AbMV in this study suggest a similar regulation by host-dependent and -specific PTMs of MP, which are needed to mediate infectivity and balance pathogenicity.

For other geminiviral proteins, phosphorylation site-dependent effects on the pathogenicity, the infectivity, or the host range have been observed as well. A point mutation in a protein kinase C recognition motif of East African cassava mosaic Cameroon virus PCP (AV2) influenced the viral pathogenicity (Chowda-Reddy et al., 2008). AC2 proteins of the two New World bipartite begomoviruses tomato golden mosaic virus (TGMV) and cabbage leaf curl virus (CaLCuV) were both phosphorylated at S109 by an upstream activating kinase of the SUCROSE-NONFERMENTING1-related kinase1 (SnRK1) in plants (Hanley-Bowdoin et al., 2013; Shen et al., 2014). Mutations introduced into the *AC2* gene at this position delayed symptom development and vDNA accumulation upon CaLCuV infection of *Arabidopsis*. S109, however, is not conserved among other (A)C2 proteins. Shen et al. (2014) discussed this as an evolutionary result in specific geminivirus host interactions, an interpretation which might apply to the results for AbMV MP here as well. The Rep of TGMV is phosphorylated at S97 by SnRK1 and its modification impaired its binding to vDNA, but not its cleavage activity (Shen et al., 2018). Interestingly, the TGMV Rep-S97D mutant showed an altered host range and did not infect tomato. This observation is in agreement with our finding that phosphorylation sites in MP influence AbMV infectivity selectively in certain host species. The TGMV Rep-S97D mutant delayed symptom development and vDNA accumulation in *N. benthamiana* considerably, but eventually, systemically infected plants displayed WT-like or even enhanced symptoms. This behavior did not correlate with vDNA contents similar to those of WT TGMV in later stages of the infection: the plants still contained only low levels of vDNA. The ßC1 protein encoded by the tomato yellow leaf curl China ß-satellite (TYLCCNB) was shown to be a substrate for SnRK1 as well (Shen et al., 2011; Shen et al., 2012; Yang et al., 2019). TYLCCNB encoding a phospho-charge-mimic mutant of ßC1 exhibited delayed symptoms and an attenuated phenotype in *N. benthamiana* upon co-infection with tomato yellow leaf curl China virus (TYLCCNV). The plants accumulated lower vDNA titers, whereas those infected with TYLCCNV/mutant TYLCCNB encoding a phosphorylation-negative alanine mutant of ßC1 contained higher vDNA titers. Nevertheless, the latter displayed symptoms resembling those of TYLCCNV/TYLCCNB WT. Such an uncoupling between symptom severity and vDNA content, as described for phosphorylation site mutants of Rep and ßC1, was observed in our previous studies for AbMV MP phosphorylation site double and single mutants as well (Kleinow et al., 2009b). For both novel triple mutants, however, the phenomenon was less pronounced. AbMV^AAA^ caused attenuated symptoms that correlated with reduced vDNA content in all infected host species. In contrast, AbMV^DDD^ induced WT-like or enhanced symptoms in *N. benthamiana*, but vDNA accumulation was transiently reduced in early stages of the infection, and increased to even higher than AbMV^WT^ titers in later stages. In the other plant species tested, AbMV^DDD^ induced milder symptoms in conjunction with lower vDNA titers, comparable to AbMV^AAA^. This suggests that host-dependent phosphorylation of a begomoviral MP can also impact pathogenicity and efficiency of infection, as detected for other geminiviral proteins before. It remains to be determined whether phosphorylation of AbMV MP affects plant defense mechanisms as suggested by prior studies of TGMV Rep and TYLCCBB βC1.

In a yeast two hybrid system, the DDD mutation abolished MP-CD self-interaction, suggesting that the negative charges introduced by phosphorylation or the respective mutants interfere with the domain’s di- or oligomerization. However, this effect was not confirmed in all layouts tested by FRET studies *in planta.* These tests indicated a similar di- or oligomerization for as for MP^AAA^ and MP^WT^. Most likely, the interaction between MP^DDD^ molecules in plant cells is rescued by the presence of other MP domains, and/or plant adapter proteins which stabilize the MP complex. Which roles MP-MP interactions play within the viral life cycle, still needs to be investigated further.

Overall, fluorescence microscopy showed subcellular distributions of both triple mutants not significantly altered in comparison to MP^WT^. This indicates that there is no fundamental role of the three phosphorylation sites in targeting MP to subcellular structures, different from some other MPs for which targeting to membranes, plasmodesmata, or nuclear-to-cytoplasmic shuttling was shown to be regulated by phosphorylation (Waigmann et al., 2000; Fujiki et al., 2006; Link et al., 2011; Nemes et al., 2017).

Upon electrophoresis in denaturing SDS polyacrylamide gels, the mobility of MP-CD^WT^ and the MP triple mutants expressed in yeast did not meet the expectations completely. In accordance with previous data (Kleinow et al., 2008; Kleinow et al., 2009b), GAD::HA::MP-CD^WT^ formed retarded extra bands, in addition to a signal at the expected molecular mass (36.7 kDa), most clearly detectable by Western blot analysis (**Figure 6C**, right panel). Interestingly, both MP triple mutants lacked signals corresponding to the expected molecular mass completely and accumulated in a number of bands with retarded migration. GBD fusion proteins behaved similarly (**Figure 6C**, left panel). GBD::MP-CD^WT^ was detected within a single band at the expected molecular mass, whereas both triple mutants showed only single bands migrating with reduced velocity. The complex band pattern of GAD::HA::MP-CD^WT^ resembles that observed for yeast-derived MP before. It was attributed to differential phosphorylation of T221 and S223 as shown by mass spectrometry, and to unidentified PTMs most likely other than phosphorylation (Kleinow et al., 2008; Kleinow et al., 2009b). The results obtained here for the two MP mutants support a presence of such unidentified PTMs reducing electrophoretic mobility. A differentially altered electrophoretic migration was observed for the FRET test proteins, too, in dependence on the fusion proteins’ layout with either N- or C-terminal position of the fluorescent protein tags. We suppose that the different layouts influence the accessibility of MP for modifying host enzymes, causing a putatively differential PTM pattern which might lead to the detected differences in electrophoretic mobility. Here, however, no differences were detectable between WT and triple full-length MP mutants. Overall, the results of this study strengthen the assumption that additional PTMs in AbMV MP still need to be uncovered.

A former phylogenetic analysis revealed that the phosphorylation site S250 is present in a highly conserved amino acid context in nearly all bipartite begomoviral MPs, whereas T221 and S223 homologues are found in New World begomoviruses only, with some variability of their exact positions (Kleinow et al., 2009b). This underscores the functional relevance of all three sites. Which plant kinases target the three phosphorylation sites in AbMV MP is an interesting question, which remains to be answered. The amino acid context of S250 does not correspond to a known kinase recognition motif. In contrast, T221 and S223 exhibit a casein kinase II (CK2) recognition motif (S/T-XX-E/D) (Meggio and Pinna, 2003). Phosphorylation of T221 generates a cluster of negatively charged amino acid residues in a region N-terminal of S223 (D-pT/D-D-S), thereby introducing a casein kinase I (CK1) recognition motif (Gross and Anderson, 1998). This suggests a control of the C-terminal oligomerization domain’s state by hierarchical phosphorylation. Members of both casein kinase types were already discovered to phosphorylate viral MPs from various viruses, including for example the +RNA-strand tobamoviruses TMV (Karpova et al., 2002; Lee et al., 2005) and ToMV (Matsushita et al., 2002; Matsushita et al., 2003), the potexvirus potato virus X (PVX) (Modena et al., 2008) and the bipartite DNA begomovirus BDMV (Lee et al., 2005). In addition, other kinases were found to be involved in the geminiviral life cycle as well, suggesting them as further candidates for MP phosphorylation. These include e.g. SnRK1, an upstream activating kinase of SnRK1, shaggy-related protein kinases and various receptor like kinases (Wang et al., 2003; Mariano et al., 2004; Wang et al., 2005; Florentino et al., 2006; Piroux et al., 2007; Shen et al., 2011; Shen et al., 2012; Hanley-Bowdoin et al., 2013; Shen et al., 2014; Shen et al., 2018). The begomoviral AC2 protein and Rep, as well as the satellite-encoded βC1 protein were uncovered to be substrates for SnRK1 (Shen et al., 2011; Shen et al., 2014; Zhong et al., 2017; Shen et al., 2018; Yang et al., 2019). These studies have indicated that phosphorylation by SnRK1 can inhibit viral protein functions, i.e. in vDNA binding for Rep, or in silencing suppression in the case of AC2 protein and ßC1, and have suggested an important role of this kinase in phosphorylation-mediated defense responses against geminivirus infections.

## Conclusion

The triple mutant MP versions of the bipartite New World begomovirus AbMV turned out to be helpful tools to investigate the functional role of plant-dependent MP phosphorylation in geminivirus–host interaction in closer detail. Our results support the assumption that AbMV MP undergoes a complex, host-dependent pattern of phosphorylation and likely dephosphorylation, and is probably embedded in a regulatory network of diverse host kinases and phosphatases controlling its phosphorylation status and thereby its effect on pathogenicity and vDNA spread, up to its successful establishment within cells. This regulatory node in the begomoviral MP may reflect host adaption aspects and impacts MP di- or oligomerization *via* its C-terminal domain. Other functions of MP may be controlled by phosphorylation as well, however, they are so far a matter of speculation. Future research will be needed to resolve the molecular details.

## Data Availability Statement

All datasets presented in this study are included in the article/**Supplementary Material**.

## Author Contributions

TK designed and coordinated the research and wrote the manuscript. AH performed research (molecular experiments, microscopy, and FRET analysis) and contributed to writing of the manuscript. SK performed research (molecular experiments, infection, and analysis of plants). LL performed research (molecular experiments, yeast two-hybrid analysis). TR performed research (molecular experiments, yeast two-hybrid analysis). JF performed research (molecular experiments, yeast two-hybrid analysis). PB performed research (molecular experiments, yeast two-hybrid analysis). GK performed research (molecular experiments, infection and analysis of plants). HJ supported research co-ordination, wrote part of and edited the manuscript. CW co-ordinated part of the research, wrote part of and edited the manuscript.

## Funding

This work was supported by the Deutsche Forschungsgemeinschaft DFG (KL1366/3-1) and by the Landesgraduierten Förderung Baden-Württemberg for AH.

## Conflict of Interest

The authors declare that the research was conducted in the absence of any commercial or financial relationships that could be construed as a potential conflict of interest.
