# Supplementary material for: Phosphorylations of the Abutilon Mosaic Virus Movement Protein Affect Its Self-Interaction, Symptom Development, Viral DNA Accumulation, and Host Range
Source: Front Plant Sci. 2020 Jul 31;11:1155. doi: 10.3389/fpls.2020.01155 (PMC7411133; doi:10.3389/fpls.2020.01155)
Supplement: Supplementary file 1 [file Presentation_1.pdf]

Supplementary Material

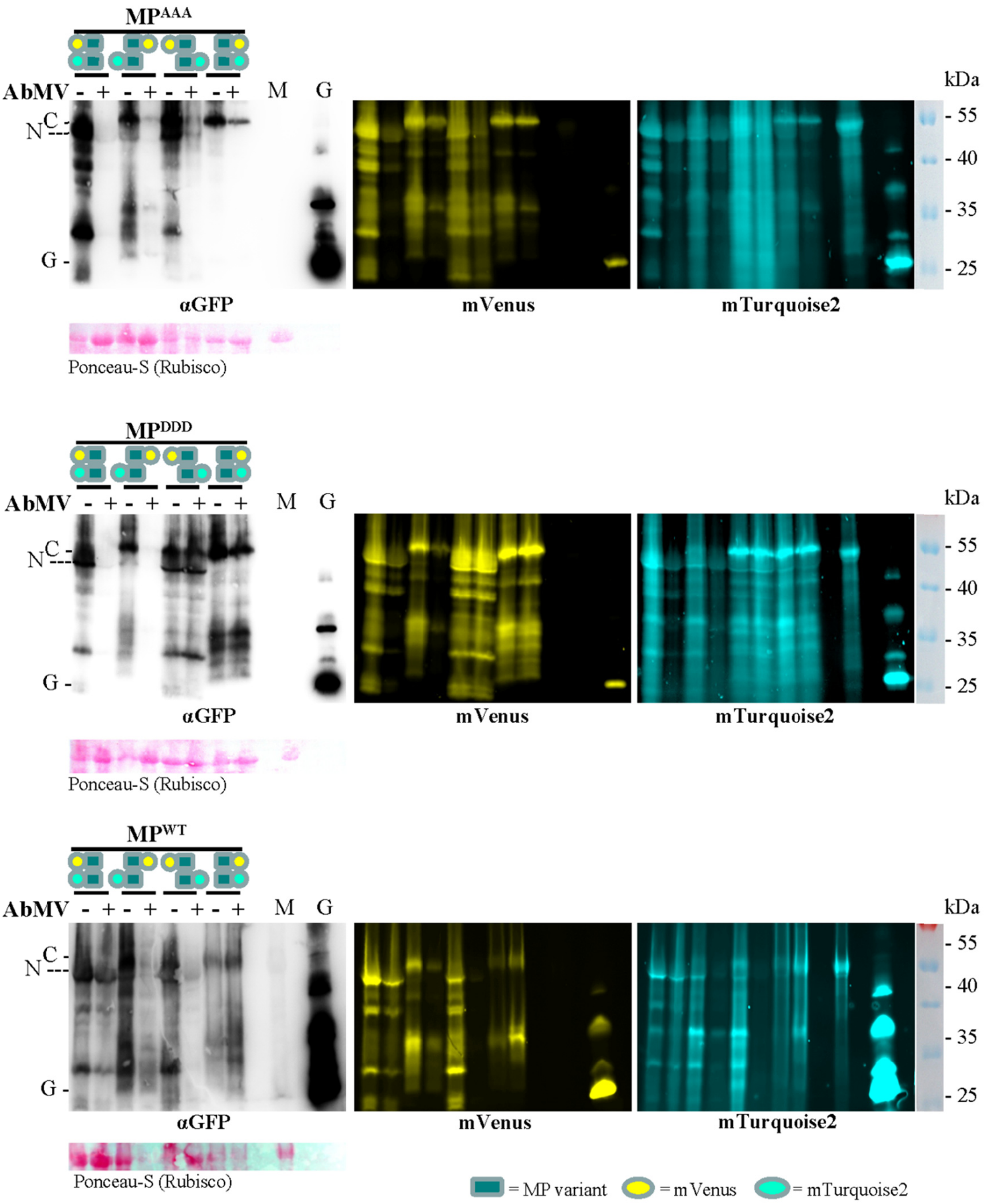

Supplementary Figure S1 Expression of FRET test proteins was confirmed by immunodetection and in-gel fluorescence monitoring of mVenus and mTurquoise2 fluorescence. Experimental set-up as described in Figure 7. Ponceau-S staining of the membrane (PonS; region of Ribulose-1, 5-

bisphosphate carboxylase/oxygenase [Rubisco] band) served as a loading control. Expected molecular mass of MP variants: C-terminally mTurquoise2 or mVenus is 61.85 kDa (C); N-terminally mTurquoise2 or mVenus is 61.82 (N); M: mock-treated control; G: positive control, purified eGFP. Please note that the combination of immunodetection and in gel-fluorescence allows the confirmation of all individual mVenus- and mTurquoise2-tagged MP versions. An unequivocal verification of $MP^{variants}$::mTurquoise2 by in in-gel fluorescence is compromised by an endogenous plant protein showing autofluorescence in the molecular weight range of C-terminally mTurquoise2-tagged MP. This was found only for SDS-PAGE conditions, whereas detection of mTurquoise2-tagged test proteins in microscopic studies was unequivocal.

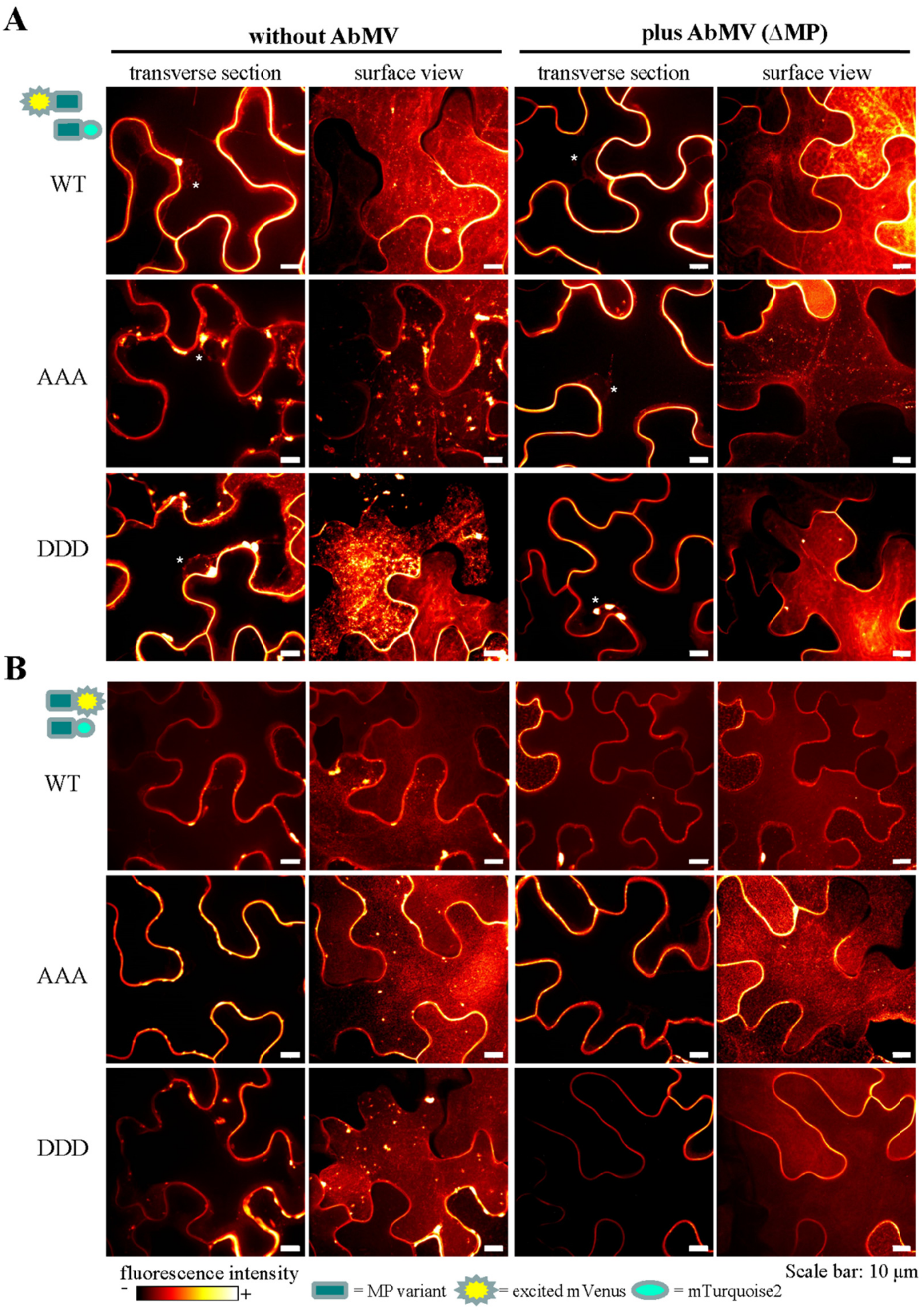

Supplementary Figure S2 Visualization of subcellular localization of MP phosphorylation site triple mutants (continued). Experimental set-up as described in Figure 8. Visualization of MP variants N- **(A)** or C-terminally **(B)** tagged with mVenus combined with the same MP type C-terminally fused to mTurquoise. Nuclei are labeled by asterisks. Scale bars: 10 µm.

Supplementary Table 1 Description of primer and templates used for cloning.

**Primer** (Sequence part corresponding to MP-encoding BC1 sequence are displayed in bold and those to added att sites for Gateway recombination cloning are underlined)	
attB1_BC1_FW	5'-ggg gac aag ttt gta caa aaa agc agg ctt a**at gga ttc tca gtt agt aaa tcc tcc** -3'
attB4_BC1-stop_REV	5'-ggg gac aac ttt gta tag aaa agt tgg gtg **ttt caa tga ttt ggc ttg aga agc c**-3'
attB4_BC1+stop_REV	5'-ggg gac aac ttt gta tag aaa agt tgg gtg **tta ttt caa tga ttt ggc ttg aga agc ctg** -3'
attB3_BC1_FW	5'-ggg gac aac ttt gta taa taa agt tgt a**at gga ttc tca gtt agt aaa tcc tcc gaa c**-3'
attB2_-stop_BC1_REV	5'-ggg gac cac ttt gta caa gaa agc tgg gtt **ttt caa tga ttt ggc ttg ag**-3'
attB2_+stop_BC1_REV	5'-ggg gac cac ttt gta caa gaa agc tgg gtt **tta ttt caa tga ttt ggc** t-3'
**PCR-Templates**	
pENTR11-BC1-CD^{WT}	BC1-CD^{WT} fragment was released via cutting with *Bam*HI and *Xho*I from pGEM-T-BC1-CD^{WT} (Frischmuth et al., 2004), and sub-cloned into the *Bam*HI and *Xho*I digested Gateway entry vector pENTR11 (Invitrogen).
pDONR207-BC1^{WT}	Krenz et al. (2010)
pDONR207-BC1^{AAA}<br>pDONR207-BC1^{DDD}	The vector pDONR207-BC1^{WT} was subjected to two rounds of a mutagenesis PCR as described in Material and Methods (2.1) to generate respective triple mutant versions of the BC1 gene.

References

Frischmuth, S., Kleinow, T., Aberle, H.J., Wege, C., Hülser, D., and Jeske, H. (2004). Yeast two-hybrid systems confirm the membrane-association and oligomerization of BC1 but do not detect an interaction of the movement proteins BC1 and BV1 of Abutilon mosaic geminivirus. *Arch Virol* 149(12)**,** 2349-2364. doi: 10.1007/s00705-004-0381-0.

Krenz, B., Windeisen, V., Wege, C., Jeske, H., and Kleinow, T. (2010). A plastid-targeted heat shock cognate 70kDa protein interacts with the Abutilon mosaic virus movement protein. Virology 401(1), 6-17. doi: 10.1016/j.virol.2010.02.011.
